# 4,6-Disubstituted pyrimidine-based microtubule affinity-regulating kinase 4 (MARK4) inhibitors: synthesis, characterization, *in-vitro* activity and *in-silico* studies

**DOI:** 10.3389/fphar.2024.1517504

**Published:** 2025-01-20

**Authors:** Ashanul Haque, Khalaf M. Alenezi, Mohd. Saeed Maulana Abdul Rasheed, Md. Ataur Rahman, Saleha Anwar, Shahzaib Ahamad, Dinesh Gupta

**Affiliations:** ^1^ Department of Chemistry, College of Science, University of Hail, Ha’il, Saudi Arabia; ^2^ Department of Biology, College of Science, University of Hail, Ha’il, Saudi Arabia; ^3^ Chemistry Program, New York University Abu Dhabi (NYUAD), Abu Dhabi, United Arab Emirates; ^4^ Centre for Interdisciplinary Research in Basic Sciences, Jamia Millia Islamia, New Delhi, India; ^5^ Translational Bioinformatics Group, International Centre for Genetic Engineering and Biotechnology (ICGEB), New Delhi, India

**Keywords:** Alzheimer’s disease, kinase inhibitors, mark 4, pyrimidine, synthesis

## Abstract

**Introduction:**

Alzheimer’s disease (AD) is a neurodegenerative disorder that significantly impacts the cognitive function and memory of a person. Despite the significant research efforts, the ability to completely prevent or effectively treat AD and its related dementias remains limited. Protein kinases are integral to AD pathology and represent promising targets for therapeutic intervention.

**Methods:**

A series of pyrimidine-based compounds 4-(4-(arylsulfonyl)piperazin-1-yl)-6-(thiophen-3-yl)pyrimidine derivatives (**8**-**14**) were synthesized and characterised. ATPase inhibition was carried out against the MARK4 enzyme. Molecular docking and molecular dynamics (MD) simulation at 500 ns was carried out against MARK4 (PDB: 5ES1). The drug-likeness feature and toxicity of the molecules were evaluated using QikProp and other tools.

**Results:**

Compounds were synthesized following a multi-step approach and characterized using multi-nuclear magnetic resonance (^1^H/^13^C-NMR) and mass spectrometry. ATPase inhibition assay of the compounds against MARK4 showed an IC_50_ value in the micromolar (μM) range. The results of the docking studies were consistent with the *in-vitro* experiments and identified (**9**) and (**14**) as the candidates with the highest affinity towards MARK4. MD simulation further supported these results, showing that the binding of ligands stabilises the target protein.

**Conclusion:**

Using experimental and theoretical approaches, we demonstrated that the reported class of pyrimidine derivatives are an excellent starting point for developing the next-generation anti-AD drugs.

## Introduction

Heterocyclic compounds are ubiquitous in both synthetic and natural chemical spaces, forming the essential backbone for a diverse array of applications (Reymond, 2015). The significance of heterocyclic compounds is immense as they are essential to humans, plants, and animals (Katritzky et al., 2010). Among wide-ranging small and medium-sized heterocycles, pyrimidine nuclei constitute an important group of pharmacologically active compounds (Das et al., 2022). The importance of this core is well supported by the fact that it serves as the fragment of nucleobases (cytosine, thymine, uracil) as well as numerous clinically approved drugs. For example, pyrimidine nucleus is present in 5-fluorouracil, imatinib (anti-cancer), rilpivirine (anti-viral), iclaprim (antibiotic), trimethoprim (anti-bacterial), and many others (Nammalwar and Bunce, 2024). Besides, its ability to serve as bioisostere (for aromatic cores) and to interact with biological targets through non-covalent interactions (NCIs) makes it an excellent candidate in drug discovery programs (Nammalwar and Bunce, 2024). A plethora of research demonstrates that pyrimidine is a promising scaffold for developing drugs against chronic and infectious diseases (Nadar and Khan, 2022). In recent years, several 4,6-disubstituted pyrimidines have been identified with anti-protozoal (Rahman et al., 2024; Singh et al., 2024), anti-inflammatory (Fatima et al., 2023), anti-neuroinflammatory (Manzoor et al., 2023) and carbonic anhydrase inhibitory (Manzoor et al., 2021a) activity.

**FIGURE 1:**
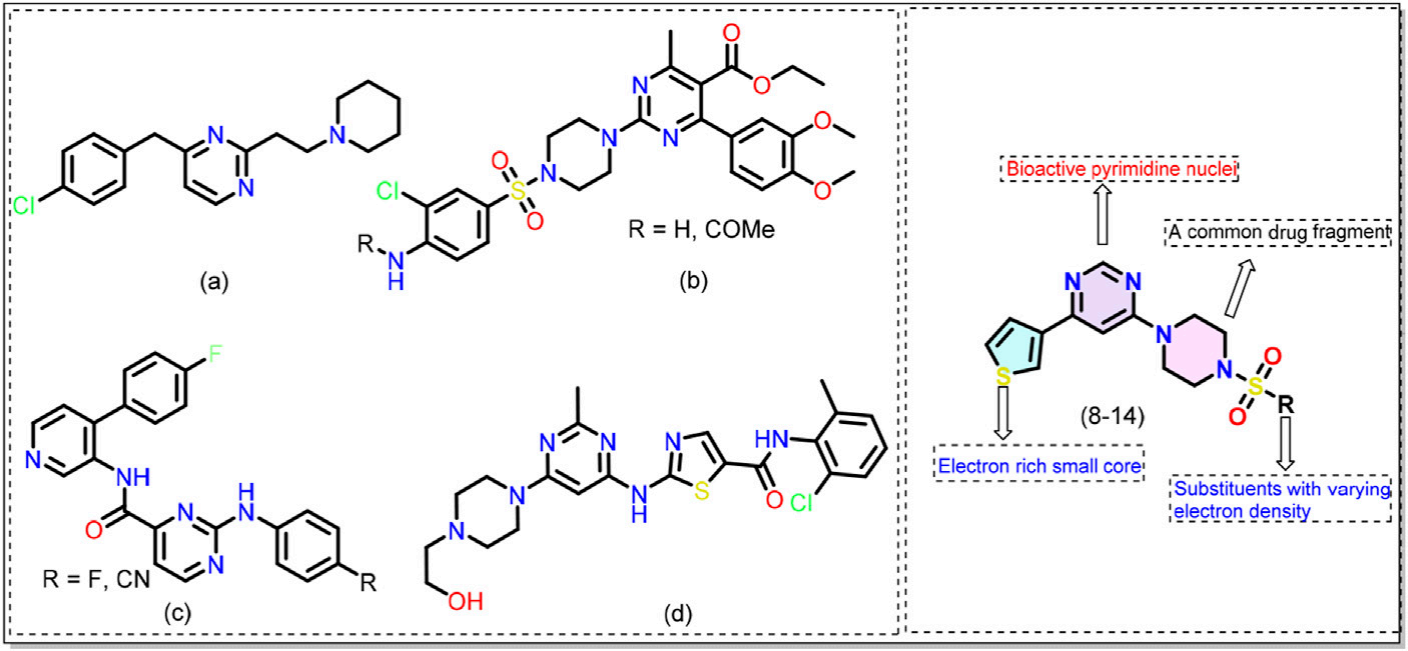
Chemical structures of previously reported pyrimidine-based kinase inhibitors (see text for more details). General structure of the 4,6-disubstituted pyrimidine derivatives (**8**-**14**) reported in this work.

Reported over a century ago, Alzheimer’s disease (AD) has now become the most widespread cause of dementia, with millions of cases reported worldwide. This has led to a significant economic and manpower burden (Bell, 2023; Gustavsson et al., 2023). The number of people suffering from AD and other dementias is estimated to surpass 152 million by 2050 (Nichols et al., 2022). To combat this debilitating condition, researchers are adopting various approaches and one of them is developing small molecules that target one or more AD machinery (e.g., β-amyloid plaques, neurofibrillary tangles) (Takahashi et al., 2017). Among different classes of small molecules identified to date, pyrimidine-based compounds emerged as a promising candidate (Singh et al., 2021; Das et al., 2022). For example, Nain and coworkers (Pant et al., 2024) reported a series of substituted pyrimidine derivatives with interesting anti-AD results. The compounds were safe at high dosages (1000 mg/kg), did not cause toxicity, and inhibited acetylcholinesterase (AChE) activity in animal models. Biological assay results indicated that the compound **a** (Figure 1) exhibits neuroprotective effects better than donepezil, a clinically used drug. By adopting a multitarget-directed ligand (MTDL) strategy, Hoda and coworkers (Manzoor et al., 2021b) reported compound b (Figure 1) that was found to be active against esterases in the nanomolar (nM) concentration. It was reported that functional groups guided the activity of the compounds with methoxy and chloro substituents showing the best activity. Since kinases contribute to the production of abnormal and hyperphosphorylated microtubule-associated tau proteins, pyrimidine-based compounds have also been studied as kinase inhibitors (Luo et al., 2016; Naz et al., 2016; Zhu et al., 2016; Jameel et al., 2017; Hartz et al., 2023). Hartz et al. (2023) reported that introducing pyrimidine provides bioactive conformation to the hinge binder and is better than the pyridine counterpart c (Figure 1). Nevertheless, the clinical success of dasatinib d (Figure 1) in CML and ALL is well known (Ayala-Aguilera et al., 2021). These studies support the idea that pyrimidine-based compounds can target multiple pathways related to Alzheimer’s disease. However, we noticed a gap, especially concerning 4,6-disubstituted pyrimidines, which have been poorly studied in relation to AD pathology, particularly regarding their interaction with kinases. Therefore, in the quest for new compounds with application in AD (Haque et al., 2024a; Haque et al., 2024b), we adopted the concept of molecular hybridization and designed hybrid compounds (**8**-**14**, Figure 1) containing a central pyrimidine ring flanked by different substituents at fourth and sixth positions. The designed molecule can be divided into three areas/regions: a fixed left-hand side electron-rich region (thiophene), and a central pyrimidine scaffold, and varying aryl (sulfonyl)piperazine fragments on the right hand. To optimize the activity of the compounds, we varied functional groups on the aromatic ring and introduced electron donor and acceptor groups of varying strength. The rationale behind the selection of these groups stems from their common use in medicinal chemistry and established bioactivity (Kilbile et al., 2023; Thakur et al., 2024). The synthesized compounds were evaluated for their activity against the microtubule affinity-regulating kinase 4 (MARK4) enzyme. This ser/thr enzyme has been reported to be over-expressed in AD and serves as the potential target for anti-AD drug development (Naz et al., 2013). The *in-vitro* biological results were complemented by molecular docking and molecular dynamics (MD) simulation studies. Attempts have been made to correlate the MARK4 activity with physicochemical characteristics, drug-likeness, toxicity, and frontier molecular orbitals of the molecules.

## Materials and methods

### General

All chemicals were procured from Sigma Aldrich and were used as received. All manipulations, except otherwise stated, were performed under a nitrogen (N_2_) atmosphere. Dichloromethane (DCM) was dried over calcium hydride (Armarego, 2017). Column chromatography was performed on silica gel (200–400 mesh) while thin layer chromatography (TLC) analysis was carried out on aluminium plates pre-coated with silica gel G_F254_. Melting points were determined by using the open capillary method and are uncorrected. ^1^H- and ^13^C-NMR spectra were recorded using a Bruker Spectrospin DPX 300 MHz spectrometer (Bruker Analytic GmbH, Berlin, Germany). The splitting patterns are denoted as follows: s for singlet, d for doublet, and m for multiplet. Chemical shift values are reported in parts per million (ppm). High-resolution mass spectrometry (HRMS) was performed using an Agilent 1290 Infinity II UPLC/HRMS instrument, equipped with a Q-TOF (UHD Accurate-Mass) and a photodiode array detector.

## General procedure for the synthesis of compounds

### Synthesis of ^t^butyl 4-(6-chloropyrimidin-4-yl)piperazine-1-carboxylate (**3**)

In a 50 mL round-bottom flask containing 15 mL of iso-propanol under N_2_ atmosphere, 4,6-dichloropyrimidine (**1**, 1.0 mmol) was added followed by 1.2 equivalents of *N*-Boc piperazine (**2**) at 0°C. To the resulting mixture, triethylamine (TEA, 1.2 mmol) was added dropwise, and the mixture was stirred. The reaction mixture was removed from the ice bath and placed at room temperature for 5–6 h and monitored by TLC. Upon the completion of the reaction, the reaction was quenched by adding 10 mL of cold water. The crude reaction mixture was extracted using DCM (3 × 30 mL) and the separated organic layer was washed with brine solution (20 mL). Afterwards, the organic layer was dried over anhyd. Sodium sulfate (Na_2_SO_4_), concentrated, and the crude product was purified using column chromatography (ethyl acetate/hexane = 8:2) to obtain intermediate ^*t*^butyl 4-(6-chloropyrimidin-4-yl)piperazine-1-carboxylate **3**.

### Synthesis of ^t^butyl 4-(6-(thiophen-3-yl) pyrimidin-4-yl)piperazine-1-carboxylate (**5**)

In a 50 mL sealed Schlenk tube ^*t*^butyl 4-(6-chloropyrimidin-4-yl)piperazine-1-carboxylate **(3,** 1.0 mmol), thiophene-boronic acid (**4,** 1.5 equiv.) and potassium carbonate (K_2_CO_3,_ 6.0 equiv.) were added. To this mixture, the solvent (8.0 mL dioxane and 2.0 mL water) was added followed by flushing with N_2_ gas for 2 min (three times). The catalyst (Pd(PPh_3_)_4_ = 5 mol%) was then added while maintaining the N_2_ atmosphere. The reaction mixture was heated at 100°C for 3 h. Upon the completion of the reaction (as confirmed by the TLC), the mixture was cooled to room temperature and the solvent was then evaporated using rotary evaporator. The resulting crude product was purified using column chromatography (ethyl acetate/hexane = 8:2) to obtain ^t^butyl 4-(6-(thiophen-3-yl)pyrimidin-4-yl)piperazine-1-carboxylate (**5**).

### Synthesis of 4-(piperazin-1-yl)-6-(thiophen-3-yl)pyrimidine **(6)**

To a 100 mL round-bottom flask containing compound **5** (1.0 mmol) dissolved in 15.0 mL DCM under a N_2_ atmosphere, trifluoroacetic acid (TFA, 1.0 mL) was added dropwise at 0°C. The reaction mixture was warmed to room temperature and stirred at room temperature for 6 h. Following the completion of the reaction, the mixture was basified using dilute sodium bicarbonate (NaHCO_3_). The organic layer was extracted with DCM (3 × 50 mL), followed by washing with water (10 mL) and brine solution (10 mL). The organic layer was dried over anhy. Na_2_SO_4_, and the solvent was evaporated using the rotary evaporator. The crude product was purified using column chromatography (DCM/MeOH = 8:3) yielding 4-(piperazin-1-yl)-6-(thiophen-3-yl)pyrimidine (**6**).

### Synthesis of 4,6-disubstituted pyrimidine derivatives **(8-14)**

In a 50 mL two-neck round-bottom flask containing 4-(piperazin-1-yl)-6-(thiophen-3-yl)-pyrimidine (**6**, 1.0 mmol) dissolved in 15 mL of dry DCM, different aryl sulfonyl chloride (**7**, 1.5 equiv.) was added slowly in portions while maintaining a nitrogen atmosphere at 0°C. Then, TEA (2.0 equiv.) was added dropwise to the mixture and stirred for 6 h. Once the reaction was complete, it was neutralized with 20 mL of a sat. NaHCO_3_ solution. The crude product was extracted using DCM (3 × 30 mL) and the combined organic layer was subsequently washed with 20 mL of cold water and 20 mL of brine solution and dried over anhyd. Na_2_SO_4_. The organic layer was evaporated on rotatory evaporator and purified by column chromatography (ethyl acetate/hexane = 8:2) to obtain **8**–**14** in moderate to good yields.

### 4-(4-((3-Chlorothiophen-2-yl)-sulfonyl)-piperazin-1-yl)-6-(thiophen-3-yl)-pyrimidine **(8)**

Synthesised as described above. White solid; yield = 78%, m. p. = 158°C; ^1^H-NMR (400 MHz, CDCl_3_) δ 8.61 (d, *J* = 1.2 Hz, 1H), 8.02 (dd, *J* = 3.0, 1.3 Hz, 1H), 7.58 (dd, *J* = 5.1, 1.3 Hz, 1H), 7.40 (dd, *J* = 5.1, 3.0 Hz, 1H), 7.34 (d, *J* = 4.0 Hz, 1H), 6.98 (d, *J* = 4.1 Hz, 1H), 6.74 (d, *J* = 1.2 Hz, 1H), 3.86 (t, *J* = 5.1 Hz, 4H), 3.18 (t, *J* = 5.1 Hz, 4H); ^13^C NMR (101 MHz, CDCl_3_) δ 162.07, 159.40, 158.52, 140.56, 138.06, 133.59, 132.19, 127.30, 126.70, 125.90, 125.68, 98.06, 45.66, 43.23. Exact mass = 426.00 amu for C_16_H_15_ClN_4_O_2_S_3_; observed mass (m/z): 427.01 amu [M + 1]^+^.

### 4-(4-((2,4-Dimethoxyphenyl)-sulfonyl)-piperazin-1-yl)-6-(thiophen-3-yl)-pyrimidine **(9)**

Synthesised as described above. Off-white solid; yield = 75%, m. p. = 172°C; ^1^H-NMR (400 MHz, CDCl_3_) δ 8.60 (d, *J* = 1.1 Hz, 1H), 8.01 (dd, *J* = 3.1, 1.3 Hz, 1H), 7.83 (d, *J* = 8.8 Hz, 1H), 7.58 (dd, *J* = 5.1, 1.3 Hz, 1H), 7.39 (dd, *J* = 5.1, 3.1 Hz, 1H), 6.74 (d, *J* = 1.2 Hz, 1H), 6.53 (dd, *J* = 8.8, 2.3 Hz, 1H), 6.48 (d, *J* = 2.3 Hz, 1H), 3.88 (s, 3H), 3.85 (s, 3H), 3.78 (t, *J* = 5.1 Hz, 4H), 3.36–3.25 (m, 4H); ^13^C NMR (101 MHz, CDCl_3_) δ 164.97, 162.25, 159.17, 158.51, 158.46, 140.67, 133.60, 126.61, 125.76, 125.72, 117.90, 104.53, 99.56, 98.08, 56.03, 55.73, 45.58, 43.96. Exact mass = 446.11 amu for C_20_H_22_N_4_O_4_S_2_; observed mass (m/z): 447.11 amu [M + 1]^+^.

### 4-(4-((4-Fluorophenyl)-sulfonyl)-piperazin-1-yl)-6-(thiophen-3-yl)-pyrimidine **(10)**

Synthesised as described above. White solid; yield = 73%, m. p. = 162°C; ^1^H-NMR (400 MHz, CDCl_3_) δ 8.59 (s, 1H), 8.00 (dd, *J* = 3.1, 1.3 Hz, 1H), 7.82–7.72 (m, 2H), 7.56 (dd, *J* = 5.1, 1.3 Hz, 1H), 7.38 (dd, *J* = 5.1, 3.0 Hz, 1H), 7.22 (m, *J* = 8.5 Hz, 2H), 6.71 (s, 1H), 3.82 (t, *J* = 5.0 Hz, 4H), 3.11 (t, *J* = 5.0 Hz, 4H); ^13^C-NMR (101 MHz, CDCl_3_) δ 166.70, 164.16, 162.07, 159.34, 158.49, 140.57, 131.48, 131.45, 130.49, 130.40, 126.67, 125.84, (d, J = 22.0 Hz), 116.70, 116.48, 98.04, 45.64, 43.34. Exact mass = 404.08 amu for C_18_H_17_FN_4_O_2_S_2_; observed mass (m/z): 405.09 amu [M + 1]^+^.

### 4-(4-((4-Nitrophenyl)sulfonyl)-piperazin-1-yl)-6-(thiophen-3-yl)pyrimidine **(11)**

Synthesised as described above. White solid; yield = 83%, m. p. = 155°C, ^1^H-NMR (400 MHz, CDCl_3_) δ 8.62 (d, J = 1.1 Hz, 1H), 8.05–7.97 (m, 2H), 7.72 (ddd, J = 7.0, 4.6, 1.9 Hz, 2H), 7.66–7.56 (m, 2H), 7.40 (dd, J = 5.1, 3.0 Hz, 1H), 6.76 (d, J = 1.2 Hz, 1H), 3.84 (t, J = 5.2 Hz, 4H), 3.43 (t, J = 5.2 Hz, 4H); ^13^C NMR (101 MHz, DMSO-d_6_) δ 162.3, 158.5, 158.4, 140.80, 135.51, 132.93, 130.87, 127.65, 126.87, 126.68, 124.67, 98.95, 45.75, 43.55. Exact mass = 431.07 amu for C_18_H_17_N_5_O_4_S_2_; observed mass (m/z): 432.08 amu [M + 1]^+^.

### 4-(4-(Pyridin-3-ylsulfonyl)-piperazin-1-yl)-6-(thiophen-3-yl)-pyrimidine **(12)**

Synthesised as described above. Light cream solid; yield = 65%, m. p. = 172°C, ^1^H-NMR (400 MHz, CDCl_3_) δ 9.01 (d, *J* = 2.4 Hz, 1H), 8.84 (d, *J* = 4.8 Hz, 1H), 8.60 (s, 1H), 8.03 (dd, *J* = 23.7, 5.6 Hz, 2H), 7.60–7.46 (m, 2H), 7.39 (dd, *J* = 5.2, 3.0 Hz, 1H), 6.72 (s, 1H), 3.85 (t, *J* = 5.1 Hz, 4H), 3.18 (t, *J* = 5.0 Hz, 4H); ^13^C NMR (101 MHz, CDCl_3_) δ 162.09, 159.44, 158.51, 153.79, 140.52, 135.34, 132.40, 126.70, 125.90, 125.65, 123.86, 98.06, 45.54, 43.37. Exact mass = 387.08 amu for C_17_H_17_N_5_O_2_S_2_; observed mass (m/z): 388.09 amu [M + 1]^+^.

### 4-((4-(6-(Thiophen-3-yl)-pyrimidin-4-yl)-piperazin-1-yl)-sulfonyl)-morpholine **(13)**

Synthesised as described above. Light color solid; yield = 85%, m. p. = 163°C, ^1^H-NMR (400 MHz, CDCl_3_) δ 8.65 (s, 1H), 8.04 (dd, *J* = 3.1, 1.3 Hz, 1H), 7.60 (dd, *J* = 5.1, 1.3 Hz, 1H), 7.41 (dd, *J* = 5.1, 3.0 Hz, 1H), 6.78 (s, 1H), 3.79 (dd, *J* = 6.4, 3.9 Hz, 4H), 3.77–3.70 (m, 4H), 3.38 (dd, *J* = 6.2, 4.0 Hz, 4H), 3.30–3.23 (m, 4H); ^13^C NMR (101 MHz, CDCl_3_) δ 162.37, 159.35, 158.51, 140.63, 126.68, 125.86, 125.71, 98.16, 66.36, 46.63, 46.12, 43.69. Exact mass = 395.11 amu for C_16_H_21_N_5_O_3_S_2_; observed mass (m/z): 396.12 amu [M +1]^+^.

### 4-(4-(Naphthalen-1-ylsulfonyl)-piperazin-1-yl)-6-(thiophen-3-yl)-pyrimidine **(14)**

Synthesised as described above. White solid; yield = 70%, m. p. = 160°C, ^1^H-NMR (400 MHz, CDCl_3_) δ 8.55 (d, *J* = 1.2 Hz, 1H), 8.34 (d, *J* = 1.8 Hz, 1H), 7.99–7.94 (m, 3H), 7.90 (dd, *J* = 8.0, 1.6 Hz, 1H), 7.74 (dd, *J* = 8.6, 1.9 Hz, 1H), 7.63 (m, *J* = 6.9, 1.5 Hz, 2H), 7.53 (dd, *J* = 5.1, 1.3 Hz, 1H), 7.36 (dd, *J* = 5.0, 3.0 Hz, 1H), 6.67 (d, *J* = 1.2 Hz, 1H), 3.94–3.75 (m, 4H), 3.17 (t, *J* = 5.1 Hz, 4H); ^13^C NMR (101 MHz, CDCl_3_) δ 162.02, 159.24, 158.44, 140.58, 135.02, 132.43, 132.21, 129.48, 129.23, 129.20, 129.09, 127.98, 127.77, 126.61, 125.78, 125.67, 122.78, 98.00, 45.77, 43.39. Exact mass = 436.10 amu for C_22_H_20_N_4_O_2_S_2_; observed mass (m/z): 437.11 amu [M + 1]^+^.

## Biological studies

### Enzyme inhibition assay

The inhibitory potential of the synthesized compounds against MARK4 was analysed using the malachite green based enzyme inhibition assay. The malachite green kinase inhibition assay is a colorimetric technique designed to assess kinase activity by detecting the release of inorganic phosphate (Pi) during phosphorylation. When a kinase transfers a phosphate group from ATP to its substrate, Pi is generated as a byproduct. The malachite green reagent interacts with Pi to produce a green-colored complex, which can be quantified using a spectrophotometer. This assay is modified to evaluate enzyme inhibition by measuring the reduction in Pi release in the presence of potential inhibitors. The concentration of ligands was gradually increased against the pure MARK4 in order to determine the inhibitory effect of the produced compounds on the kinase activity of MARK4. The protein’s highest activity, MARK4 (ligand-free), was utilised as a reference, and various ligand concentrations were added to determine the IC_50_ value. MARK4 (5 μM) and freshly made ATP were combined, and a final volume (100 μL) of the reaction mixture was created. It was then incubated for 1 hour at 25°C. To stop the reaction, we added 200 μL of Malachite green solution to the mixture. After that, we incubated the samples at room temperature to allow the color to develop. Finally 96 well plates were filled with 100 μL of the reaction mixture, which was then scanned at 620 nm using a multiscan ELISA reader. All the reactions were carried out in triplicates.

## Computational studies

### Modeling of MARK4 structure

The 3D structure of the MARK4 protein (PDB ID: 5ES1) was downloaded from the protein data bank (Sack et al., 2016). The crystal structure of MARK4 lacks amino acid residues between 205 and 218. Therefore, it was modelled prior to the docking and simulation studies. The missing residues were reconstructed using a web-based SWISS-Model server (Schwede et al., 2003). The sequence of the missing region was input into the SWISS-Model server, which used homologous structures to predict and build the missing loop. The quality of the modeled region was assessed by comparing it to the original crystal structure through structural alignment and validation techniques, such as a Ramachandran plot. The combined structure, incorporating both the crystallographic data and the modeled residues, was used for further computational studies.

### Ligand and target preparation

The 2D chemical structures were drawn and converted to 3D using ChemDraw. Energy minimisation and geometry optimisation were then performed to ensure stable conformations. Non-ionic form of the ligands was employed in the docking studies. Input files were prepared using AutoDock Tools (ADT, v1.5.7) and the docking was performed using AutoDock Vina (v1.1.2) (Huey et al., 2012). Docking was performed using a grid box of dimension 70 × 88 × 84 Å^3^, a default grid spacing of 0.375 Å and default parameters for the rest of the variables. The output file produced were analysed in PyMol (DeLano, 2002). A 2D interaction plot of the best-docked conformation was generated using Schrödinger Visualizer (Schrödinger Release 2024-4: Maestro, Schrödinger, LLC, New York, NY, 2024).

### Molecular dynamics (MD) simulation

MD simulations of the docked ligand-MARK4 complexes were conducted using the Desmond module in Schrödinger (Bowers et al., 2006). Initially, the docked ligand and target MARK4 complexes were merged within the Maestro interface (Friesner et al., 2006). The system setup involved embedding the merged complex into an orthorhombic water box, ensuring a 10 Å buffer distance from the protein. The water was modeled using the TIP4P model, which provides a realistic representation of water’s properties in MD simulations (Release, 2017). To achieve electrostatic neutrality, counterions (Na⁺/Cl⁻) were added. This step is crucial for maintaining proper ionic strength and mimicking physiological conditions within the simulation environment. The OPLS_2005 force field was employed to describe atomic interactions within the system accurately (Shivakumar et al., 2010). The ensemble class was set to NPT (constant pressure and temperature) with the temperature maintained at 300 K using the Nose-Hoover thermostat, while the pressure was stabilized and at 1 atm using the Martyna-Tobias-Klein barostat (Lippert et al., 2013). A pre-production equilibration phase lasting 10 ns was performed, during which the system’s trajectory was recorded at intervals of 4.8 ps to monitor initial adjustments. Following equilibration, a production run of 500 ns was executed to explore the dynamic stability of the ligand-MARK4 complex comprehensively. Post-simulation analysis involved calculating the root mean square deviation (RMSD), root mean square fluctuation (RMSF) and evaluating secondary structure elements (SSE) along with protein-ligand interaction plots.

### Drug-likeness, bioavailability, and toxicity prediction

To evaluate the physicochemical and pharmacokinetic properties of the compounds, we employed both free and commercial computational tools, each offering unique strengths. For freely accessible resources, we used SwissADME (Daina et al., 2017) and pKCSM (Pires et al., 2015), for predicting drug-likeness, solubility, absorption, and other key pharmacokinetic parameters. SwissADME provides insights into lipophilicity, water solubility, and bioavailability, while pKCSM models complex ADMET (absorption, distribution, metabolism, excretion, and toxicity) properties, enhancing our understanding of the compounds’ safety and efficacy profiles. For a more refined analysis, we used the commercially available QikProp (Shahbazi et al., 2017; Omoboyowa, 2022), which offers high-precision predictions of molecular properties and ADMET profiles.

## Density functional theory (DFT) study

Quantum chemical calculations were performed using Spartan 20 software (Hehre and Huang, 1995) using the B3LYP/6-31G* level of theory. This DFT analysis yielded chemical descriptors, including total energy (E), chemical hardness (η), chemical potential (μ), and electrophilicity (ω).

## Results and discussion

### Synthesis and characterization of 4-(4-(arylsulfonyl)piperazin-1-yl)-6-(thiophen-3-yl)pyrimidine derivatives (**8**-**14**)

**SCHEME 1:**
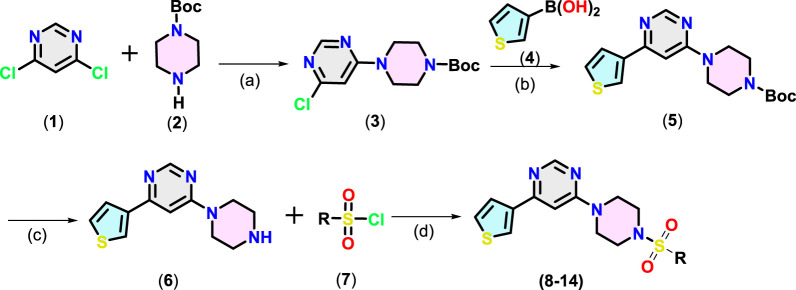
Synthesis of pyrimidine derivatives (**8**-**14**). Reaction conditions: (a): 1 (1.0 mmol), 2 (1.2 equiv.), isopropanol, TEA, 0°C to RT, 5-6 h, (b): 3 (1.0 mmol), 4 (1.5 equiv.), K_2_CO_3_ (6.0 equiv.), dioxane/H_2_O (8:2), 100°C, 3 h, (c): 5 (1.0 mmol), TFA (1.0 mL), 0°C to RT, 6 h (d) 6 (1.0 mmol), 7 (1.5 equiv.), Et_3_N (2 equiv.), 0°C to RT, 6 h

Target compounds 4-(4-aryl piperazin-1-yl)-6-(thiophen-3-yl)pyrimidine derivatives (**8–14**) were synthesised following the steps given in Scheme 1. Nucleophilic aromatic substitution (S_N_Ar) reaction between 4,6-dichloropyrimidine (**1**) with the *tert*-butoxycarbonyl (Boc)-protected piperazine (**2**) in the presence of triethylamine (TEA) in iso-propanol afforded Boc-protected intermediate (**3**). Suzuki–Miyaura coupling between 3-thienylboronic acid (**4**) and (**3**) using Pd(PPh_3_)_4_ catalyst and K_2_CO_3_ as the base in aqueous dioxane under reflux afforded (**5**). Finally, Boc was deprotected using trifluoroacetic acid (TFA) in dichloromethane (DCM). The resulting product (**6**) underwent reaction with different substituted arylsulfonyl chloride (**7**) using TEA in DCM to afford 4-(4-(arylsulfonyl)piperazin-1-yl)-6-(thiophen-3-yl)pyrimidine derivatives (**8–14**) in 65%–80% yield.

**FIGURE 2:**
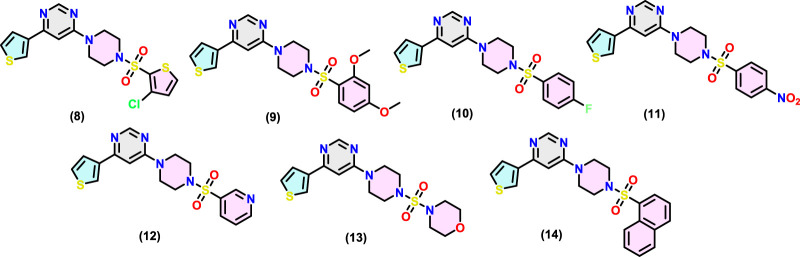
Chemical structures of MARK4 inhibitors (**8**-**14**).

The chemical structure of the final compounds (Figure 2) was confirmed by proton (^1^H-NMR), carbon (^13^C-NMR), and mass spectrometry. All spectral data are given in Supplementary Figures SF1–SF7 (*supporting information*). Characteristics resonances observed in ^1^H-NMR include signals for the piperidine ring, thienyl ring, methylene of piperazine, and aromatic core attached to it through sulfonyl core. For instance, the ^1^H-NMR spectrum of (**8–14**) showed multiplet at δ 3.17–3.43 ppm and δ 3.38–3.94 ppm integrated for eight hydrogens of piperazine core. Two protons of the pyrimidine ring appeared at δ 7.36-7.58 and δ 8.59–8.65 ppm (Gong et al., 2017). Another characteristic signal (e.g., methoxy protons at δ 3.85–3.88 ppm as a singlet in compound 9) equalled the number of different protons and are in agreement with the formula. Similarly, in ^13^C-NMR, carbon of the aromatic rings and others was observed at expected chemical shift values. For instance, the ^13^C-NMR spectrum of (**8–14**) shows a characteristic peak for piperazine at δ 43.23–43.96 ppm and δ 45.54–46.12 ppm. In the case of compound (**9**), two carbons resonating at δ 66.36 ppm and δ 46.63 ppm can be attributed to *C*-O and *C*-N, respectively of the morpholine moiety. Finally, the MS results further provided the identification of compounds. All compounds showed (M + 1) molecular ion peaks at m/z 427.01, 447.11, 405.09, 432.08, 388.09, 396.12 and 437.11 amu for (**8–14)**, respectively, confirming formulations for the final compounds.

## Biological assay

### Enzyme (MARK4) inhibition assay

**FIGURE 3:**
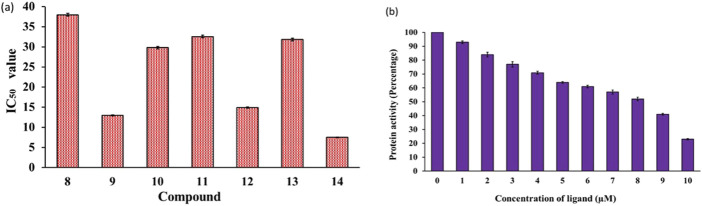
**(A)** Enzyme inhibition assay of compounds (**8–14**) and **(B)** variation of MARK4 activity with the ligand (**14**) concentration.

MARKs, or microtubule-affinity regulating kinases, are a group of serine/threonine kinases found in mammals and other organisms (Naz et al., 2013). These kinases play a critical role in phosphorylating microtubule-associated proteins, which then go on to regulate important processes like cell cycle progression and cytoskeletal dynamics. These kinases are quite abundant in the human brain and are known to be key players in the development and progression of various diseases including cancer and ADs (Alam et al., 2023). Despite their high importance, there has been limited research on the development of synthetic small molecules (inhibitors) against MARK4 (Li et al., 2020; Voura et al., 2022). In the present study, we selected MARK4 as the target and assessed our compounds against it. The kinase inhibitory potential of the compounds against MARK4 was examined using an ATPase inhibition assay (Yousuf et al., 2015; Yousuf et al., 2022). Figure 3 displays the kinase activity profile of the inhibitors (**8**-**14**) against MARK4. We found that compounds with electron rich aryl substituents exhibited relatively higher activity. For example, compound (**14**), with naphthyl substituents, exhibited the best activity (IC_50_ = 7.52 ± 0.33 μM) followed by (**9)** (IC_50_ = 12.98 ± 0.63, Figure 3A) containing 2,4-dimethoxyphenyl substituent. However, the other five compounds displayed relatively lower activity with IC_50_ between 14.92 ± 0.53-37.99 ± 0.62 μM (activity order: **14** > **9** > **12** > **10** > **13** > **11** > **8**). It is also notable that as the concentration of compound (**14**) increases, the activity of MARK4 decreases significantly (Figure 3B), underscoring their potential as MARK4 inhibitors. Following this, attempts have been made to collect the fluorescence quenching of MARK4 in the presence of the best inhibitor (**14**). However, to our surprise, it did not show appreciable quenching of the emission (*data not included*). The rationale behind the behaviour is currently under investigation.

## *In-silico* assay

### Molecular modeling and validation

**FIGURE 4:**
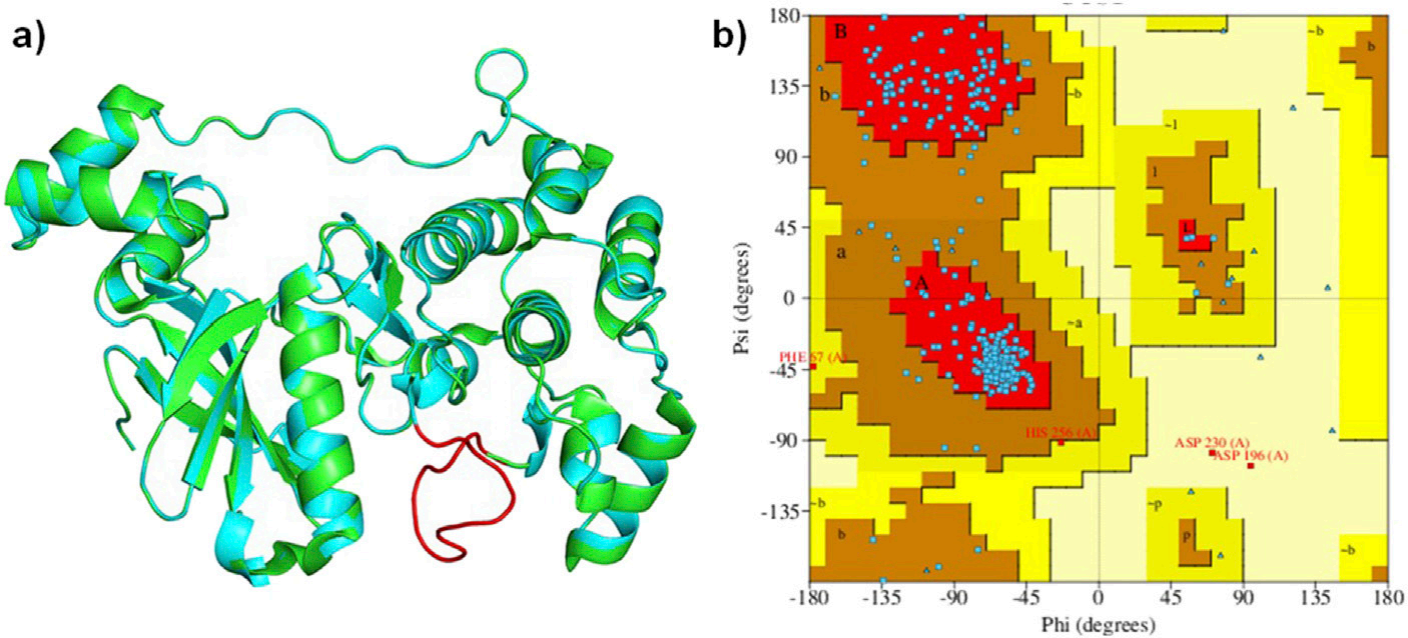
**(A)**: Ribbon diagram displaying the superimposed MARK4 (PDB ID: 5ES1) alongside the modeled MARK4, and **(B)** the Ramachandran plot of the modeled structure.

The analysis of the MARK4 crystal structure (PDB ID: 5ES1) indicated some missing regions (205-218) in the available structure. Therefore, using 5ES1 as a template and the web-based platform SWISS-Model server, missing residues were modeled (Figure 4A). The accuracy of the modeled structure was verified using a Ramachandran plot. The analysis revealed that 85.9% of the residues are located in the most favored regions, 12.6% in the additional allowed regions, 0.7% in the generously allowed regions, and only 0.7% in the disallowed regions (Figure 4B). The modeled structure showed that the structure is reliable for further studies.

### Molecular docking

**FIGURE 5:**
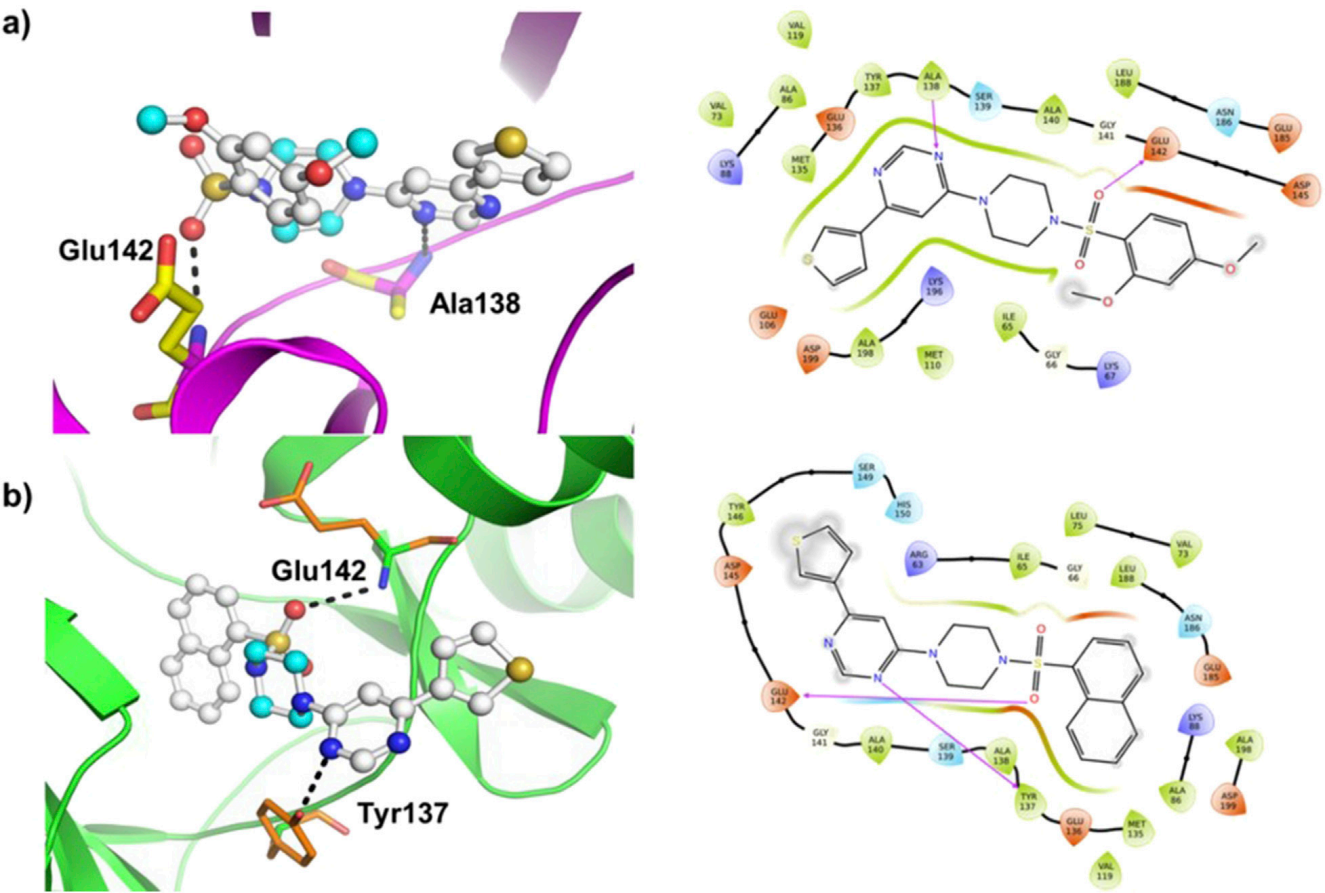
Molecular docking results of **(A)** ligand (**9**) and **(B)** ligand (**14**) with the MARK4 enzyme (PDB: 5ES1). The protein is depicted as a cartoon model, while the ligands are shown in a ball-and-stick representation.

Molecular docking was carried out using AutoDock Vina program (v1.1.2) using compounds (**8–14**) as the ligands and MARK4 receptor (PDB: 5ES1) as the target. The findings of the study are shown in Figure 5 and Supplementary Figure SF8 (*supporting information*). The binding energy of the compounds (Table 1) varies slightly as compounds share high structural similarity. Among the studied series, compound (**14**) demonstrated the highest binding score (ΔG = −8.3 kcal/mol) and interacted through H-bonds with Tyr137 and Glu142 residues, as well as hydrophobic interactions. Compound (**10**) also showed similar value (ΔG = −8.3 kcal/mol), establishing H-bonds with Lys67 and Glu185 residues of MARK4. Compounds (**11)** and (**12**) had a slightly lower values (ΔG = −8.2 kcal/mol for both), despite interacting with different partners in H-bonding. Additionally, compounds (**9**) (ΔG = −8.0 kcal/mol), (**8**) (ΔG = −7.6 kcal/mol), and (**13)** (ΔG = −7.3 kcal/mol) displayed appreciable binding affinity and formed H-bonds with residues such as Ala138, Glu142, and Tyr137 (Supplementary Figure SF8).

**TABLE 1:** The docking study results of MARK4 with the studied compounds.

Compounds	(8)	(9)	(10)	(11)	(12)	(13)	(14)
Binding energy (kcal/mol)	−7.6	−8.0	−8.3	−8.2	−8.2	−7.3	−8.3
H-bonding residues	Ala138	Ala138, Glu142	Lys67, Glu185	Ala138, Asp199	Lys67, Glu185	Ala138	Tyr137, Glu142

## MD simulation

### Root mean square deviation (RMSD) and root mean fluctuation (RMSF) analysis

**FIGURE 6:**
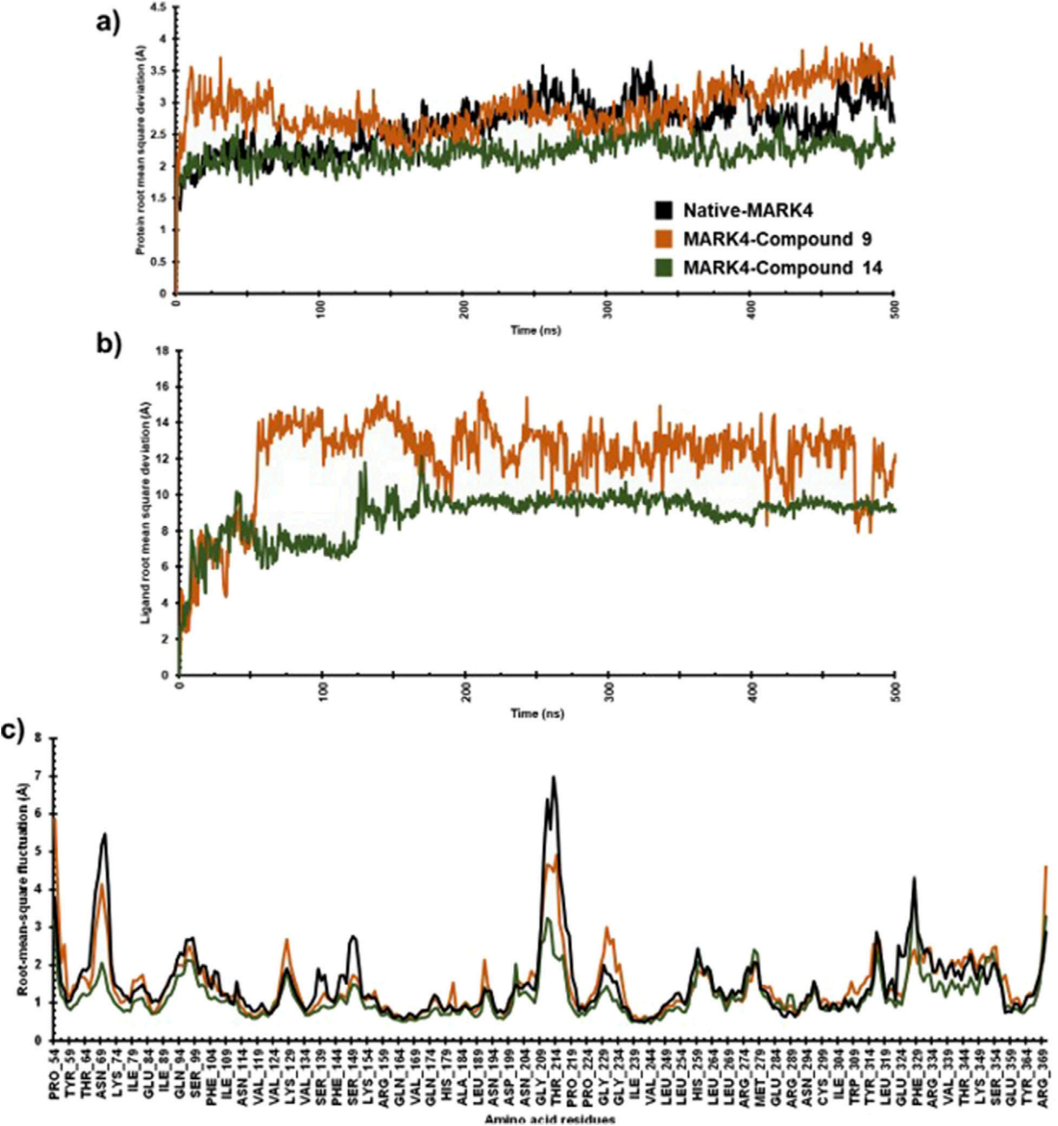
The protein RMSD **(A)**, ligand RMSD **(B)**, and RMSF **(C)** plots comparing the native MARK4 structure with its complexes with compounds (**9**) and (**14**).

To assess the stability and dynamic characteristics of the MARK4 protein-ligand complexes, molecular dynamics (MD) simulation at 500 ns scale was carried out for the complexes containing compounds (**9**) and (**14**). The stability profiles were assessed through RMSD and RMSF analysis, comparing the native MARK4 structure and its complexes with the two ligands. The RMSD result provides insights into the overall conformational stability of the protein-ligand complex (Du et al., 2016; Ahamad et al., 2024), while the RMSF highlights the flexibility of individual residues throughout the simulation (Martínez, 2015), contributing to a thorough understanding of the dynamic behaviour of the complex. The RMSD plot indicated that the native MARK4 and its complex with compound (**9**) reached equilibrium within the first 25 ns of the simulation (Figure 6A). However, the same complex showed significant fluctuations between 25 and 75 ns, with an RMSD value peaking at 3.5 Å. The inspection of the trajectory indicated that it stabilized between 75 ns and 200 ns with minimal fluctuations. Beyond 200 ns, an increase in RMSD fluctuations was observed, indicating a less stable complex. In contrast, the MD trajectory of compound (**14**) with MARK4 exhibited a different pattern. Up to 125 ns, the RMSD of the (**14**)-MARK4 complex closely resembled that of the native MARK4 structure. After 125 ns, some deviations were noted, but the trajectory for compound (**14**) remained stable throughout the simulation time, showing fewer fluctuations and better stability than compound (**9**). The average RMSD values of the native MARK4 and (**9**)-MARK4 complex were approximately 2.64 Å and 2.91 Å, respectively. On the other hand, the (**14**)-MARK4 complex maintained a lower average RMSD of 2.21 Å, indicating greater structural stability. These findings suggest that compound (**14**) forms a more stable complex with the MARK4 receptor compared to compound (**9**), maintaining better equilibrium and fewer deviations throughout the simulation period. The lower average RMSD of the compound (**14**) underscores its potential to stabilize the MARK4 protein structure more effectively, thereby enhancing its role as a promising MARK4 inhibitor. The MD simulation results indicate that the average RMSD values for ligands (9) and (14) in complex with the MARK4 protein were 11.97 Å and 8.85 Å, respectively (Figure 6B). Notably, compound (**14**) demonstrated greater structural stability within the MARK4 binding pocket, as indicated by its lower RMSD compared to compound (**9**). This lower RMSD suggests that compound (**14**) maintains a more stable binding conformation over the course of the simulation, highlighting its potential as a MARK4 inhibitor.

For both the native MARK4 and the (**9**)-MARK4 complex, several regions (Thr64-Lys74, Asn204-Pro219, Pro224-Gly234, Glu324-Phe329, Tyr364-Arg369) showed fluctuations. These regions, particularly in loops and surface-exposed areas, exhibited notable flexibility, potentially indicating instability or conformational changes affecting the overall structural integrity of the protein. In contrast, the (**14**)-MARK4 complex demonstrated reduced fluctuations across these regions, suggesting that compound (**14**) effectively stabilized the protein structure. The lower RMSF values indicate that it restricts the flexibility of key residues, contributing to a more rigid and stable conformation of the MARK4 protein compared to the native structure and the complex with compound (**9**). The RMSF values were calculated for the native MARK4 protein, as well as for the MARK4 complexes with compounds (**9**) and (**14**). The RMSF values for the native MARK4, (**9**)-MARK4, and (**14**)-MARK4 complexes were approximately 1.46 Å, 1.52 Å, and 1.15 Å, respectively (Figure 6C). The higher RMSF value in the (**9**)-MARK4 complex indicates tighter binding at specific sites but with higher flexibility in other regions. On the other hand, the (**14**)-MARK4 complex demonstrates a balanced profile, providing stability across the protein structure. Consequently, the RMSF analysis strongly supports the finding that compound (**14**) offers greater stabilization to the MARK4 receptor, limiting the flexibility of key regions and maintaining a more consistent structural conformation throughout the simulation.

### Secondary structure elements (SSE)

**FIGURE 7:**
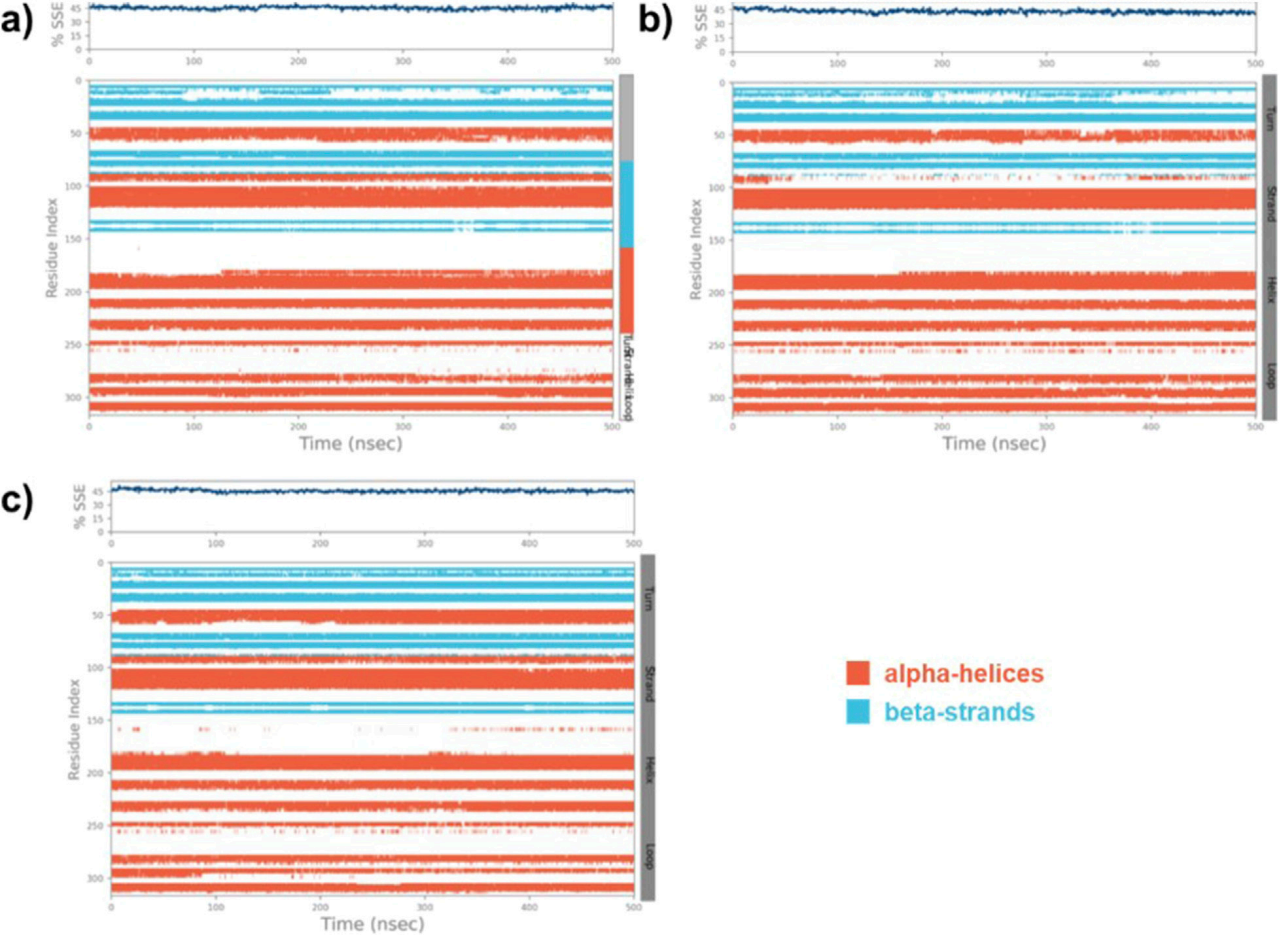
The SSE plot of the native MARK4 **(A)** and its complexes with compounds (**9**) and (**14**) **(B, C)**, displays the distribution of SSE by residue index and their composition over time.

The SSE of the native MARK4, (**9**)-MARK4, and (**14**)-MARK4 complexes were evaluated to analyse the structural integrity and stability of the enzyme in the presence of ligands. The SSE was calculated as the percentage of helix and strand content, which are crucial indicators of stability (Swain et al., 2023). For the native MARK4 protein, the SSE values were calculated to be 45.08% SSE (31.97% helix and 13.11% strand) (Figure 7A). This reflects the inherent structural stability of the protein in its unbound state, with a balanced distribution between α-helical and β-strand regions. In the case of the (**9**)-MARK4 complex, the SSE values were slightly reduced (SSE = 43.18%) with 30.44% helix and 12.74% strand (Figure 7B). A decrease in helical and strand content suggests that compound (**9**) induces subtle conformational changes in the protein, leading to destabilisation, particularly in regions critical for maintaining the protein’s overall fold (Daggett, 2006). In contrast, the (**14**)-MARK4 complex exhibited improved structural stability with 45.64% SSE (31.51% helix and 14.13% strand) (Figure 7C). These values are higher than those observed for the **9**-MARK4 complex and the native MARK4, indicating that compound (**14**) preserves and even enhances the SSEs of the MARK4 receptor. The increased strand content in the presence of compound (**14**) is a significant factor contributing to the overall stability of the protein-ligand complex. The analysis of SSE confirms that compound (**14**) stabilises the MARK4 protein more effectively compared to compound (**9**), maintaining a robust secondary structure throughout the MD simulation.

**FIGURE 8:**
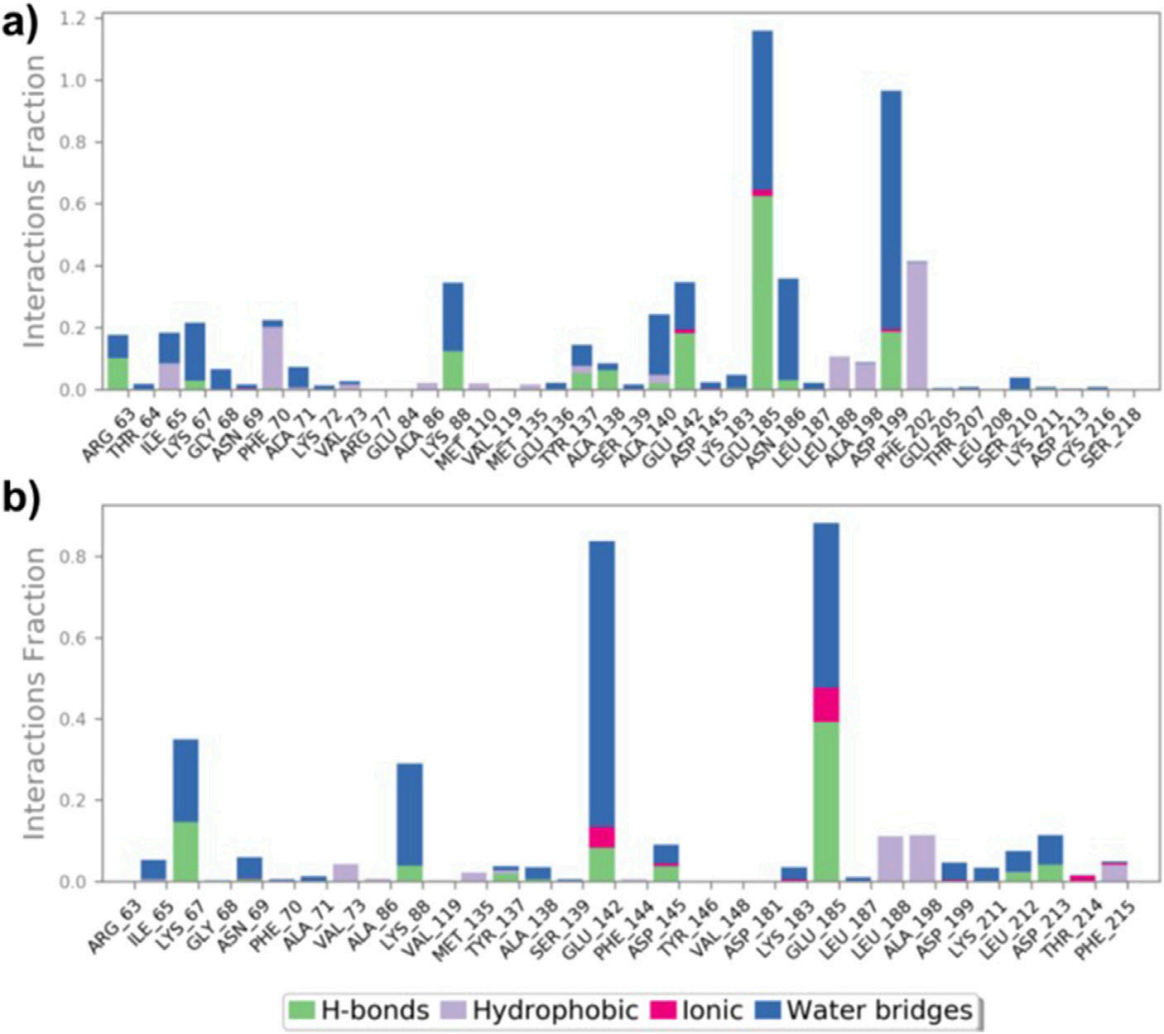
Interactions (hydrogen bonds, hydrophobic, ionic, and water bridges) between MARK4 and ligands (**9**) and (**14**) throughout the MD simulation **(A, B)**.

Protein-ligand interactions during simulations of (**9**)-MARK4 and (**14**)-MARK4 complexes were monitored. Key interactions, including H-bonds, hydrophobic interactions, ionic interactions, and water bridges, were the main contributors throughout the simulation period (Figure 8). Analysis of the ligand (**9**)-MARK4 complex during the simulation revealed stabilizing interactions, particularly with the following residues: Arg63, Ile65, Lys88, Thr137, Ala138, Ala140, Glu185, Asn186, and Asp199 (Figure 8A). (Kristiansen, 2004) These residues play an important role in maintaining binding within the active site pocket. Similarly, the MD simulation of the ligand (**14**)-MARK4 complex demonstrated H-bonding with residues Lys67, Lys88, Thr137, Glu142, Asp145, Lys211, and Asp213. This underscores their significance in anchoring the ligand within the active site.

## Pharmacokinetic properties

To assess whether a compound is suitable for pharmaceutical use, it is essential to evaluate several critical factors. These include the drug-likeness and examination of its absorption, distribution, metabolism, excretion, and toxicity (ADMET) properties. Such evaluation helps determine the possible fate of the compound within the body, ensuring it can be safely and effectively utilized in medical treatments (Jia et al., 2020). Besides, it also aids researchers in guiding the design of new molecules with desired characteristics and establishing correlations with biological outcomes (Wager et al., 2016). Following the molecular docking and dynamics studies, we assessed the drug-likeness, bioavailability, and toxicity profiles of compounds (**8–14**). Table 2 presents some of the results obtained from SwissADME (Ritchie et al., 2011). It is quite clear that the compounds under investigation align well with Lipinski’s drug-likeness parameters (Lipinski et al., 2012), with all compounds exhibiting TPSA within the defined range of 20–130 Å (Daina and Zoete, 2016). The top three compounds, (**14,** TPSA = 103 Ǻ), (**9,** TPSA = 121.5 Ǻ), and (**12,** TPSA = 115.9 Ǻ), which displayed lower TPSA values, also exhibited low IC_50_ values. Conversely, compounds (**11,** TPSA = 148.8 Ǻ) and (**8**, TPSA = 131.3 Ǻ) with higher TPSA values showed higher IC_50_ values. We observed an inverse relationship between activity and the log*P* value, suggesting that the compounds may be interacting with the receptor through hydrophobic interactions.

**TABLE 2:** Drug properties of compounds (**8**-**14)**. For details, see the text.

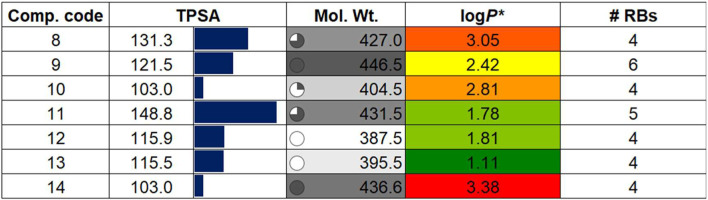

Additional drug-like properties of the compounds were studied using the QikProp (Schrödinger) tool (Shahbazi et al., 2017; Omoboyowa, 2022). This tool is valuable for calculating various drug-likeness parameters, especially for CNS drug development (Ghose et al., 2012). The results (Tables 3, 4) obtained aligned well with both experimental and computed values discussed earlier. For instance, the compounds exhibited 2-4 rotatable bonds (within the limit of 0–15) and dipole moments ranging from 2.82 to 8.22, falling within the specified range of 1–12.5. Additionally, none of the compounds deviated from Ro5/Ro3 parameters. Furthermore, the CNS activity predicted using this tool indicated that all compounds, with the exception of (**11**) (CNS = −2), displayed moderate activity towards the CNS (CNS = −1 to 0). This is a crucial consideration for drugs intended for CNS applications. This compound also has highest also exhibited the highest TPSA value and relatively lower human oral absorption (%HOA). The compounds screened showed similar values for total solvent-accessible surface area (SASA = 623.85-706.02) and related hydrophobic (FOSA = 117.66-303.64), hydrophilic (FISA = 69.58-180.20), π (PISA = 194.66-440.07), and weakly (55.96-150.39) surface area components (Haque et al., 2024a; Haque et al., 2024b). The octanol/water partition coefficient (QPlog*P*o/w) for the compounds evaluated fell within the generally accepted range of −2.0 to 6.5, with measured values spanning from 2.09 to 4.28. Additionally, the negative brain–blood partition coefficient (QPlogBB value) was acceptable for all the compounds except (**11**) (QPlogBB = −1.35), which was the lowest among all. Also, compound (**11**) showed the lowest Caco-2 permeability rate (good if > 500 nm s^−1^), indicating its weak drug-likeliness (Nunes et al., 2013). The results of the toxicity prediction studies (Supplementary Table ST1, *supporting information*) (Pires et al., 2015) reveal that compounds (**8**), (**11**), and (**12**)exhibited AMES toxicity, while compound (**14**), identified as the most active within the series, displayed the highest maximum tolerated dose (MTD = 0.308 log mg/kg/day). Additionally, none of the compounds were anticipated to act as inhibitors of hERG I/II inhibitor, except for compound (**10**), which demonstrated potential as an hERG II inhibitor. While the compounds are not expected to induce skin sensitization, there is a possibility of hepatotoxicity. It is essential to emphasize that the aforementioned predicted values are projected, and further research is necessary to obtain an accurate representation.

**TABLE 3:** Drug-likeness features of the compounds determined using the QikProp.

Code #	#Rotor	CNS	Dipole	SASA	FOSA	FISA	PISA	WPSA	Volume	HBD	HBA	RO5/RO3
**(8)**	2	0	6.46	651.22	122.38	81.98	296.47	150.39	1148.87	0	7	0/0
**(9)**	4	−1	8.22	706.02	303.31	93.18	253.09	56.45	1282.21	0	8.5	0/0
**(10)**	2	0	4.72	649.50	117.66	94.99	332.05	104.80	1150.02	0	7	0/0
**(11)**	3	−2	2.82	687.35	129.86	180.20	321.32	55.97	1214.53	0	8	0/0
**(12)**	2	−1	6.11	637.33	129.14	107.55	344.68	55.96	1125.62	0	8.5	0/0
**(13)**	2	−1	4.37	623.85	303.64	69.58	194.66	55.96	1120.87	0	9.2	0/0
**(14)**	2	0	7.16	688.86	117.68	74.40	440.07	56.71	1253.95	0	7	0/0

**TABLE 4:** The ADMET properties of the studied compounds.

Code #	QPpolrz	QPlog PC16	QPlog Poct	QPlog Pw	QPlog Po/w	QPlog S	CIQP logS	QPlog HERG	QPP Caco	QPlog BB	QPPM DCK	QPlog Kp	QPlog Khsa	%HOA
**(8)**	41.37	12.61	18.56	10.37	3.74	−5.22	−5.95	−5.95	1653.76	−0.01	5679.77	−1.79	−0.004	100
**(9)**	45.01	12.99	20.34	11.28	3.39	−4.74	−5.73	−5.84	1295.02	−0.49	1333.36	−1.96	−0.103	100
**(10)**	41.76	12.01	18.37	10.68	3.52	−4.99	−5.54	−6.07	1244.70	−0.25	2350.74	−1.91	−0.001	100
**(11)**	43.60	13.46	19.46	12.02	2.67	−4.81	−5.65	−6.29	193.66	−1.35	169.94	−3.42	−0.124	83.49
**(12)**	40.90	12.24	18.92	12.28	2.37	−3.76	−4.48	−6.07	946.24	−0.48	944.03	−2.09	−0.473	94.10
**(13)**	39.28	10.89	18.13	11.66	2.09	−3.14	−4.06	−5.11	2167.93	−0.08	2312.81	−1.92	−0.674	100
**(14)**	46.95	13.90	20.35	11.35	4.28	−5.53	−6.46	−6.60	1951.35	−0.16	2083.71	−1.15	0.301	100

## Density functional theory (DFT) study

Density functional theory (DFT) calculation is a routine but important technique used to understand structural, chemical, photo-physical, and many other underlying properties at atomic and molecular levels (Van Mourik et al., 2014). For example, chemical hardness, based on the HOMO-LUMO energy gap, reflects stability and resistance to charge transfer in molecules. These descriptors help understand molecular electronic properties and potential reactivity.

**FIGURE 9:**
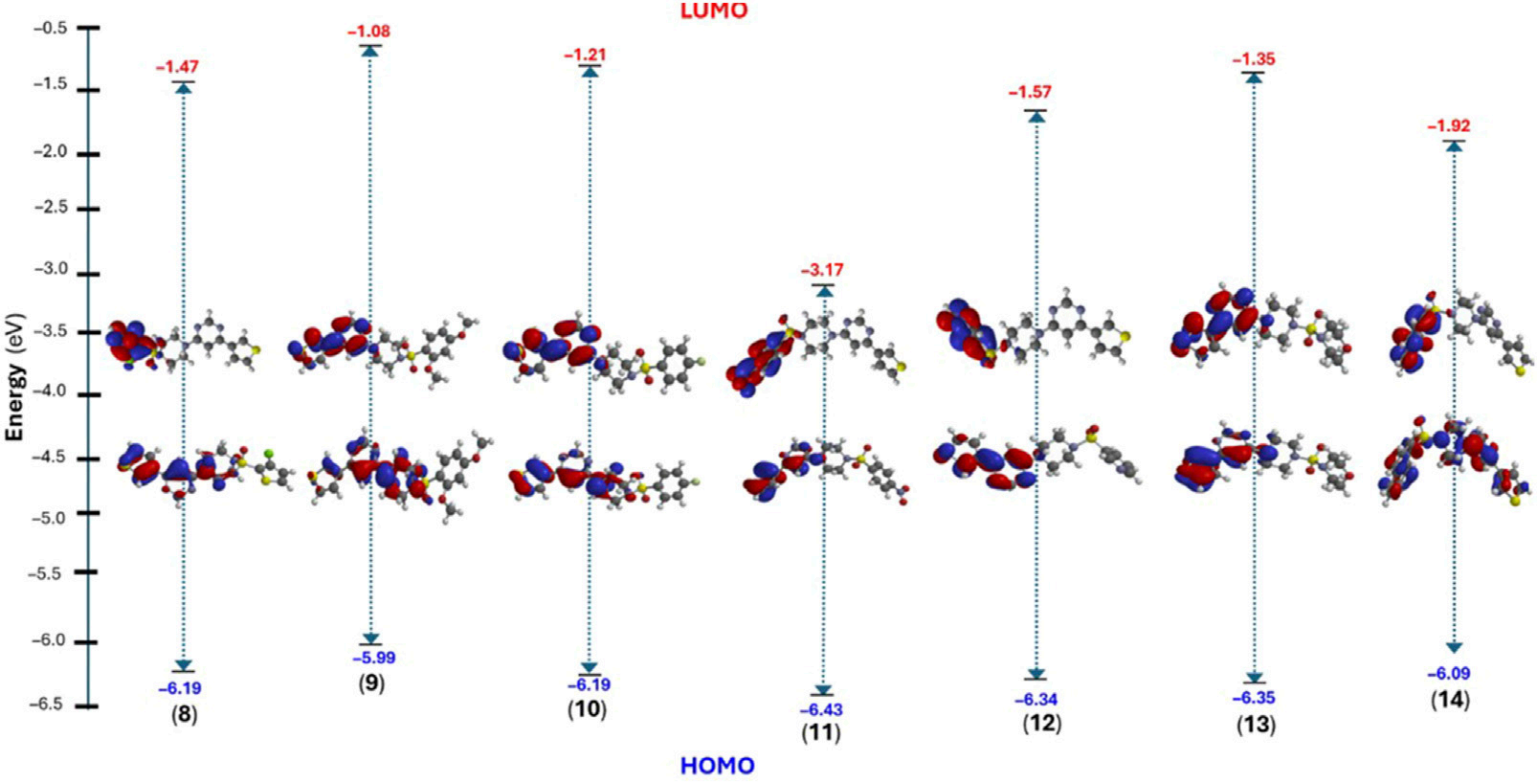
DFT calculated frontier molecular orbitals (HOMO/LUMO) energy level and their distribution in compounds (**8–14**).

Considering this, we performed structural optimization followed by frontier orbital calculations for the compounds (**8**-**14**). All calculations were performed using B3LYP/6-31G* in the gas phase, and the results are depicted in Figure 9, Supplementary Figure SF9, and Supplementary Table ST2 (*supporting information*). The energy levels of the highest occupied molecular orbital (E_HOMO_) ranged from −5.99 to −6.43 eV, while the lowest unoccupied molecular orbital (E_LUMO_) levels were observed between −1.08 and −3.17 eV. This resulted in a band gap (ΔE) between 3.26 and 5.00 eV, which is consistent with previously small molecules (Arshad et al., 2022; Guerraoui et al., 2023). Figure 9 shows that HOMO was localized over the 4-thienyl group with little contribution from the pyrimidine core. On the other hand, the 6-aryl group contributed to the LUMO only, and piperazine served as the conjugation break. This observation clearly explains why there were no major changes in the HOMO energy levels while LUMO level varied with change in aryl group. The introduction of a strong electron-withdrawing nitro group significantly deepens the LUMO (−3.17 eV) level with considerable change in the HOMO level (−6.43 eV), too, leading to the smallest energy gap (ΔE = 3.26 eV). It is also notable that the two most active compounds also had higher HOMO levels.

Once the energy level of the molecules is in hand, we then proceed to compute chemical descriptors as they can be correlated with the biological activity of the molecule and underpins several properties of a molecule (Sánchez-Bojorge et al., 2009; Kawakami et al., 2013; Mushtaque et al., 2016; Frau and Glossman-Mitnik, 2017). For example, the HOMO energy level of a molecule impacts its electron-donating ability, with higher levels indicating greater ability. A smaller ΔE value corresponds to a lower energy requirement for electronic excitation, resulting in increased chemical reactivity and softer molecule (Mermer et al., 2020). According to the data obtained, the ionization energy (*I*) ranged between 5.99 and 6.43 eV while the electron affinity (*A*) of the compounds were in the range of 1.08–3.17 eV. Other descriptors, such as negative μ value (−2 to −4.80 eV) and reasonable ω (0.85–7.06 eV) indicate the stable nature of the molecule (Parr et al., 1999). Finally, the electrostatic potential (ESP) map showed the expected distribution of the electron-deficient and rich regions over carbocycles and heteroatoms, respectively.

## Conclusion

The development of new drug candidates for application in chronic diseases such as cancer, diabetes, and AD continues to be an exciting area of research. In this paper, we presented the chemical and biological properties of a series of 4,6-disubstituted pyrimidine-based small molecules. The synthesised compounds have been synthesized and fully characterised using NMR and MS spectrometry. Final compounds were then assessed *in-vitro* against the MARK4 enzyme, which has emerged as a potential target to combat neurodegenerative diseases. The biological findings were supported by computational studies, including molecular docking, MD simulation, and ADME/T analysis. We demonstrated that compounds are efficient against MARK4, and the binding of the most potent compound (**14**) stabilises the protein. The results of the MD simulation studies indicated that compound (**14**) showed reduced RMSD and RMSF value while enhancing the SSE, especially strands. This enhanced stability could play a crucial role in the efficacy of compound (**14**) as a potential inhibitor of MARK4, making it a promising candidate for further drug development. We also found that, to some extent, the activity of the compound is directly proportional to the TPSA while inversely proportional to the log*P* values.

## Data Availability

The original contributions presented in the study are included in the article/Supplementary Material, further inquiries can be directed to the corresponding author.
