# Supplementary material for: 4,6-Disubstituted pyrimidine-based microtubule affinity-regulating kinase 4 (MARK4) inhibitors: synthesis, characterization, in-vitro activity and in-silico studies
Source: Front Pharmacol. 2025 Jan 20;15:1517504. doi: 10.3389/fphar.2024.1517504 (PMC11788324; doi:10.3389/fphar.2024.1517504)
Supplement: Supplementary file 1 [file DataSheet1.pdf]

4,6-Disubstituted pyrimidine-based microtubule affinity-regulating kinase 4 (MARK4) inhibitors: Synthesis, characterization, in-vitro activity and in-silico studies

Ashanul Haque,[1,*] Khalaf M. Alenezi,[1] Mohd. Saeed Maulana Abdul Rasheed[2], Md. Ataur Rahman[3], Saleha Anwar[4], Shahzaib Ahamed,[5] Dinesh Gupta[5]

[1] Department of Chemistry, College of Science, University of Hail, Kingdom of Saudi Arabia.

[2] Department of Biology, College of Science, University of Hail, Kingdom of Saudi Arabia.

[3] Chemistry Program, New York University Abu Dhabi (NYUAD), Saadiyat Island, United Arab Emirates.

[4] Centre for Interdisciplinary Research in Basic Sciences, New Delhi, India

[5] Translational Bioinformatics Group, International Centre for Genetic Engineering and Biotechnology (ICGEB), Aruna Asaf Ali Marg 110067 New Delhi, India

Supporting information

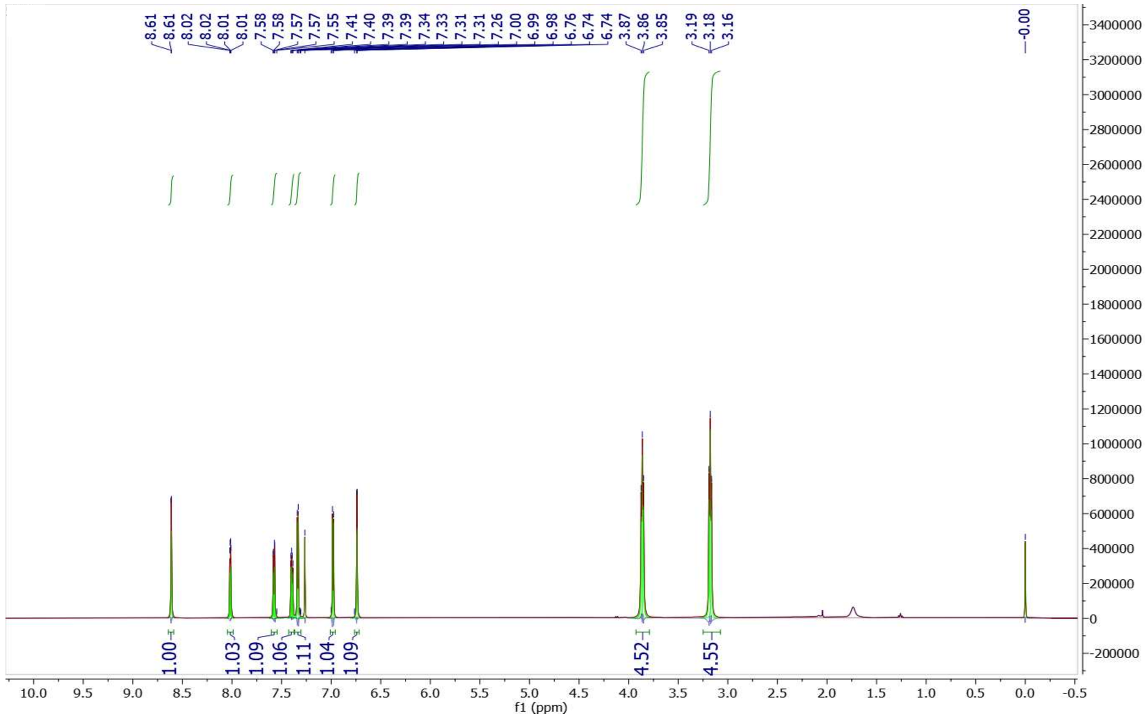

8.61 8.61 8.02 8.02 8.01 7.58 7.58 7.57 7.57 7.55 7.41 7.40 7.39 7.39 7.34 7.33 7.31 7.31 7.26 7.00 6.99 6.98 6.76 6.74 6.74 3.87 3.86 3.85 3.19 3.18 3.16
-0.00
1.00 1.03 1.09 1.06 1.11 1.04 1.09 4.52 4.55
f1 (ppm)

(a)

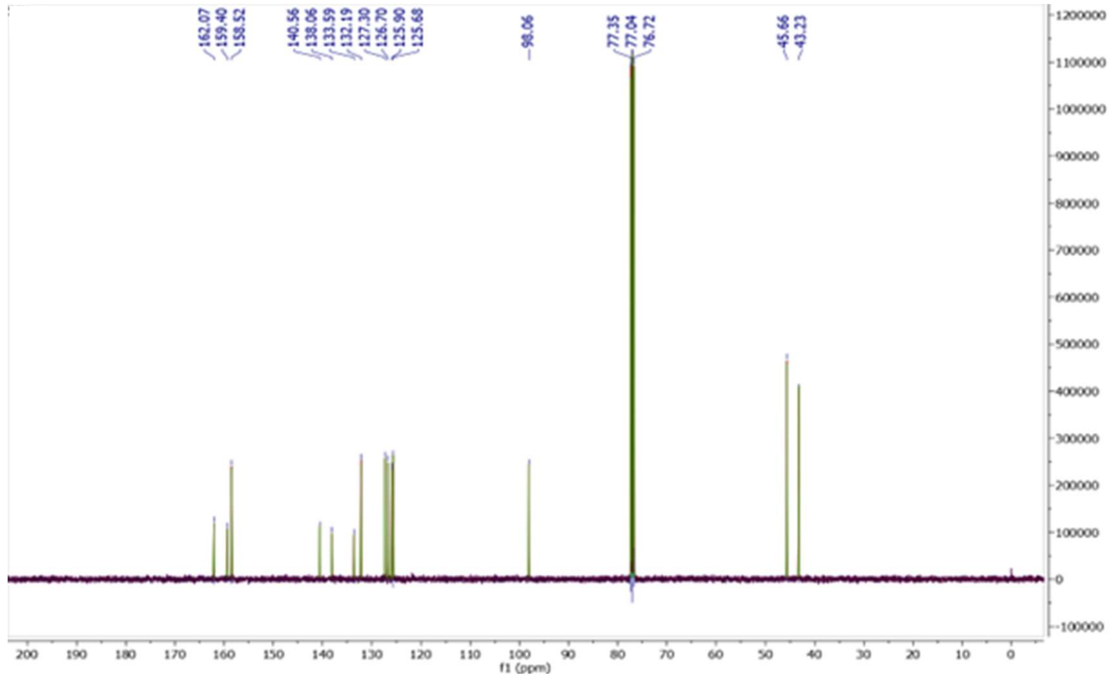

162.07 159.40 158.52 140.56 138.06 133.59 132.19 127.30 126.70 125.90 125.68
98.06
77.35 77.04 76.72
45.66 43.23
f1 (ppm)

(b)

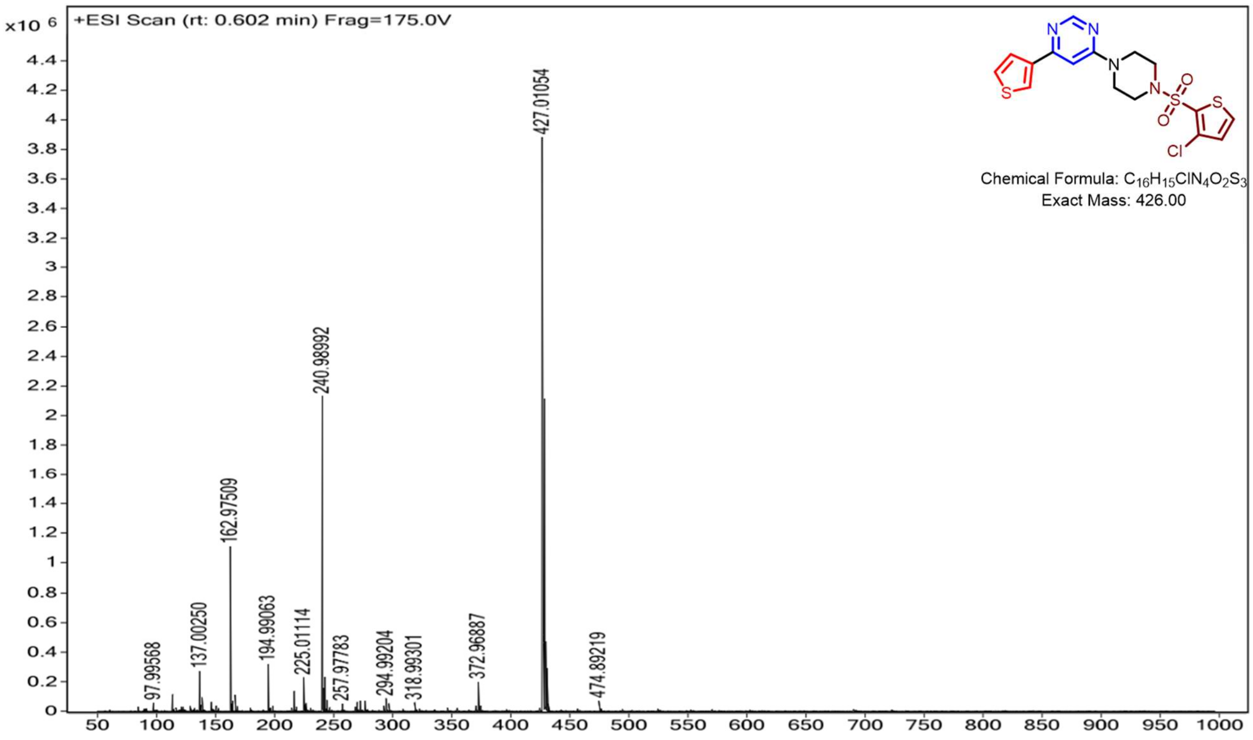

(c)

Figure SF1: (a) ^{1}H-NMR, (b) ^{13}C NMR and (c) Mass spectra of **8**.

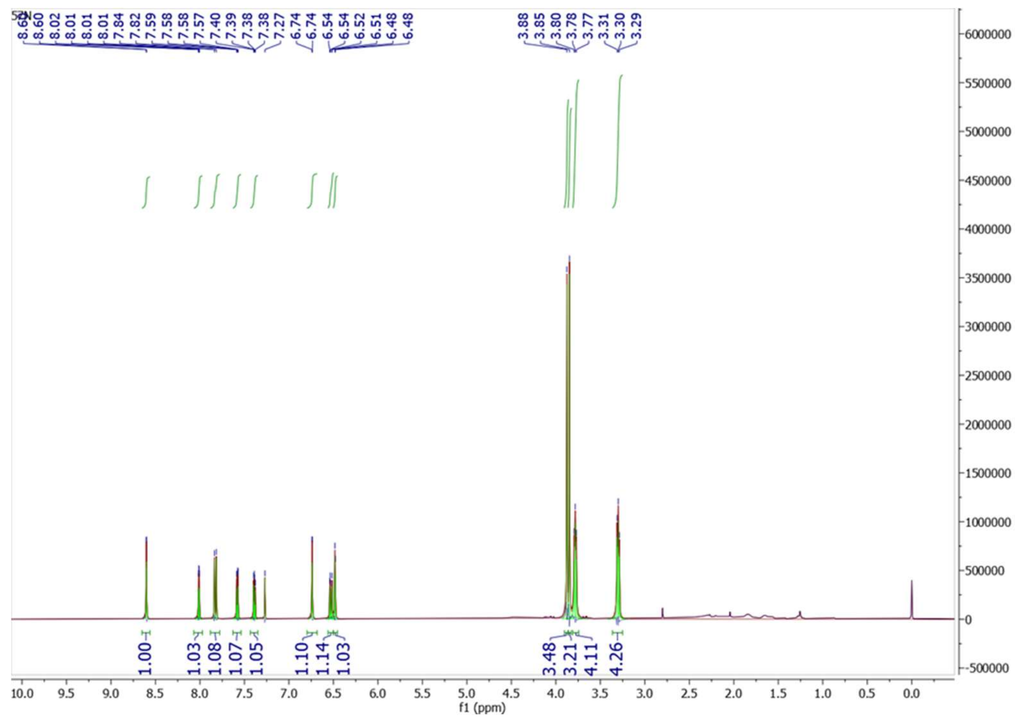
52N
8.60
8.60
8.02
8.01
8.01
8.01
7.84
7.82
7.59
7.58
7.58
7.57
7.40
7.39
7.38
7.38
7.27
6.74
6.74
6.54
6.54
6.52
6.51
6.48
6.48
3.88
3.85
3.80
3.78
3.77
3.31
3.30
3.29
1.00
1.03
1.08
1.07
1.05
1.10
1.14
1.03
3.48
3.21
4.11
4.26
f1 (ppm)

(a)

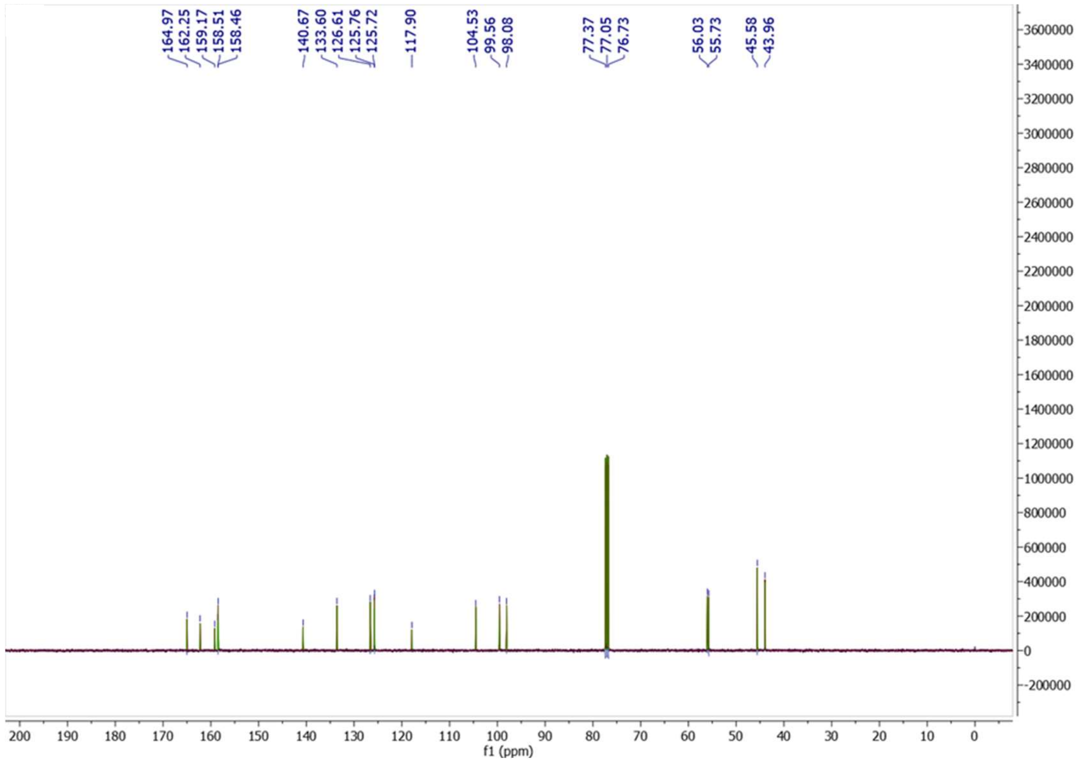
164.97
162.25
159.17
158.51
158.46
140.67
133.60
126.61
125.76
125.72
117.90
104.53
99.56
98.08
77.37
77.05
76.73
56.03
55.73
45.58
43.96
f1 (ppm)

(b)

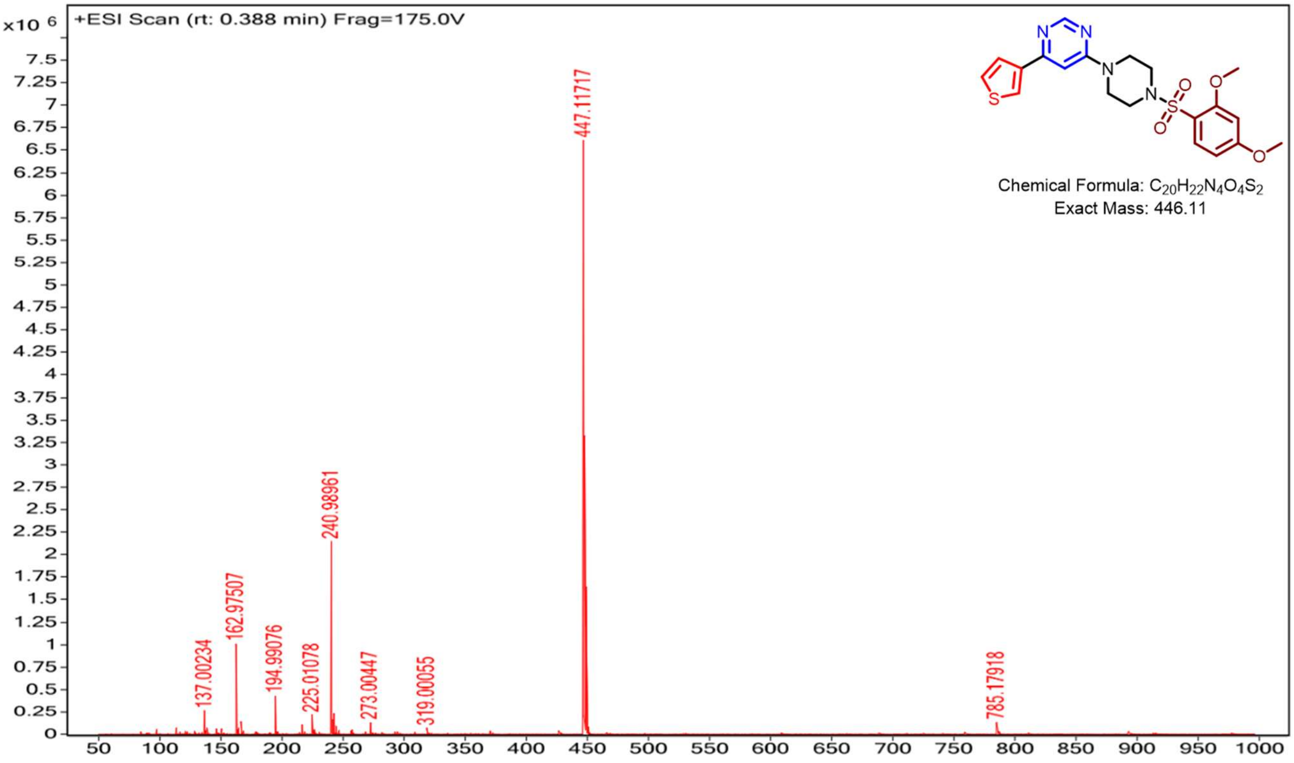

(c)

Figure SF2: (a) ^{1}H-NMR, (b) ^{13}C NMR and (c) Mass spectra of **9**.

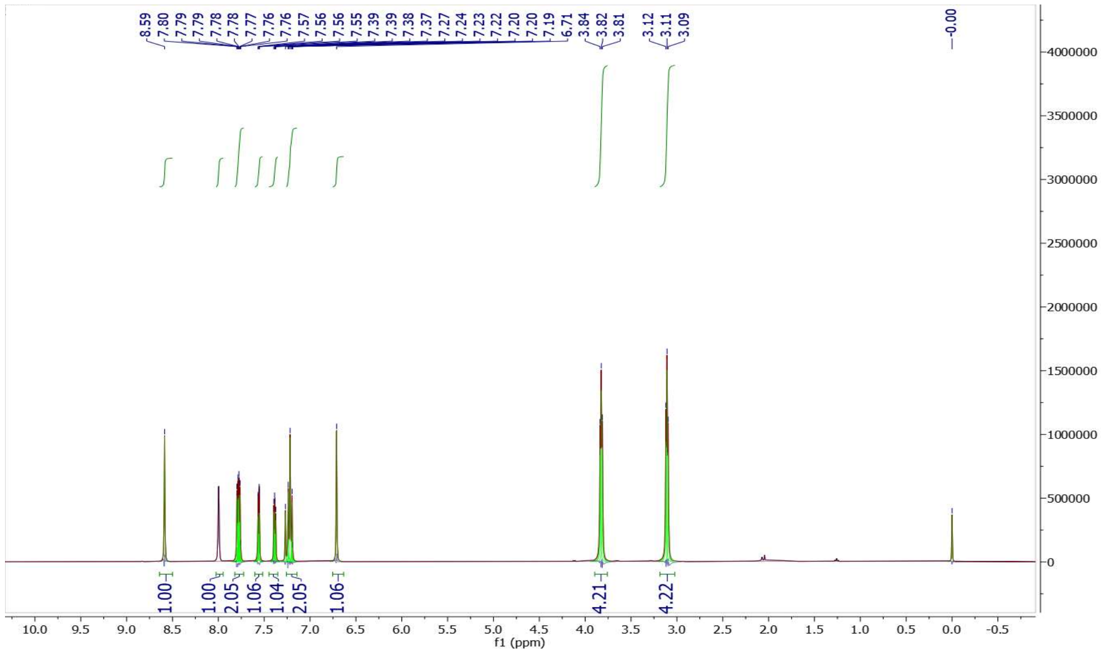
8.59
7.80
7.79
7.79
7.78
7.78
7.77
7.76
7.76
7.57
7.56
7.56
7.55
7.39
7.39
7.38
7.37
7.27
7.24
7.23
7.22
7.20
7.20
7.19
6.71
3.84
3.82
3.81
3.12
3.11
3.09
-0.00
1.00
1.00
2.05
1.06
1.04
2.05
1.06
4.21
4.22
f1 (ppm)

(a)

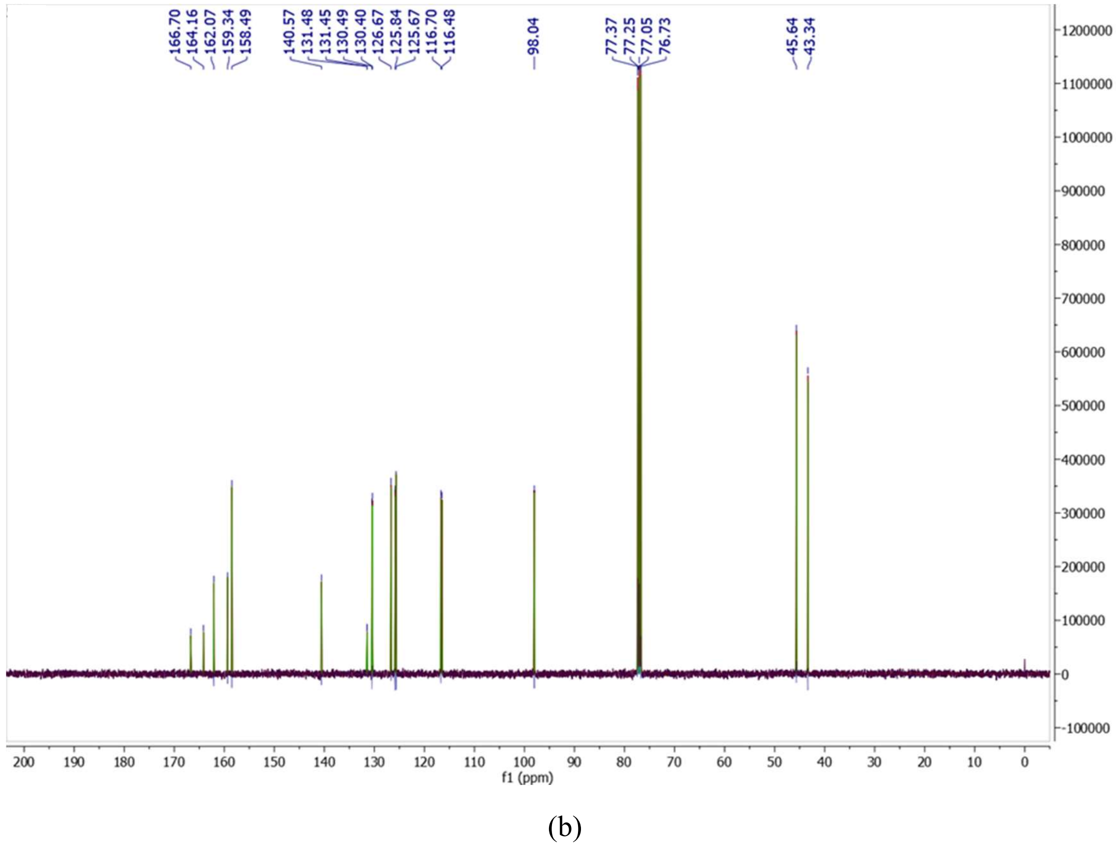
166.70
164.16
162.07
159.34
158.49
140.57
131.48
131.45
130.49
130.40
126.67
125.84
125.67
116.70
116.48
98.04
77.37
77.25
77.05
76.73
45.64
43.34
f1 (ppm)

(b)

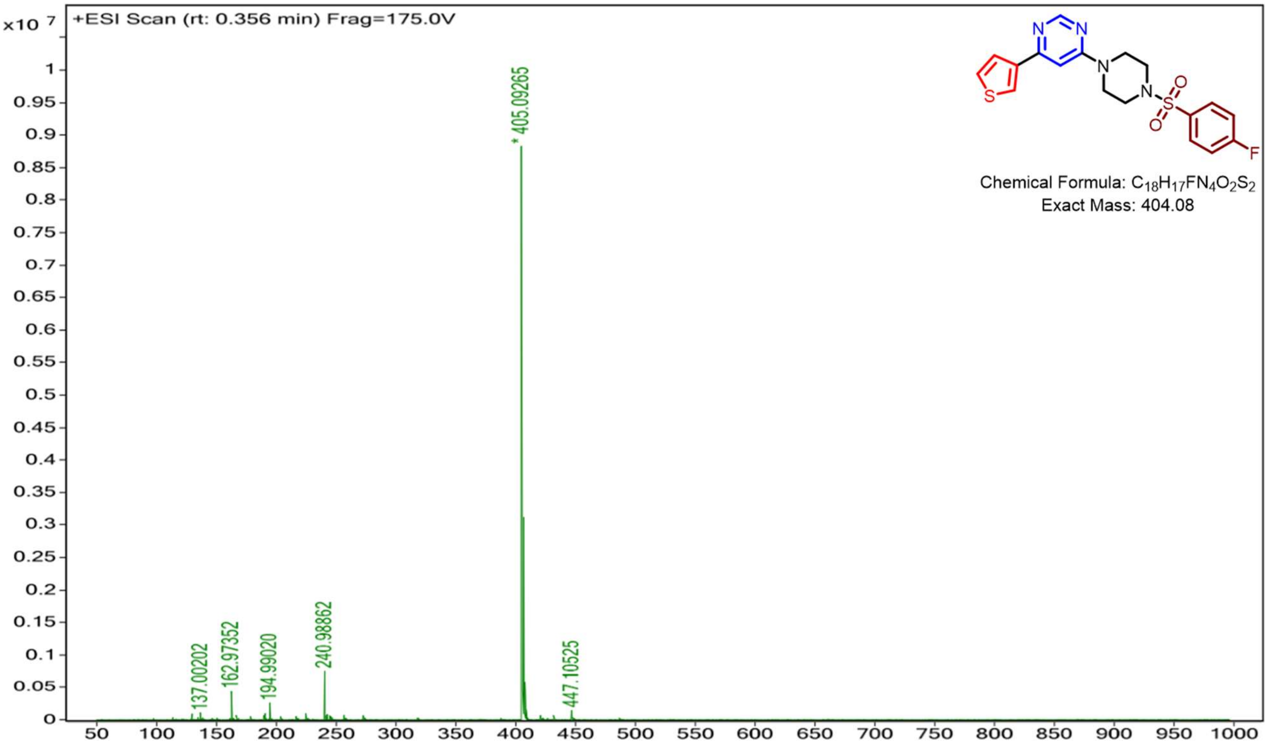

(c)

Figure SF3: (a) ^{1}H-NMR, (b) ^{13}C NMR and (c) Mass spectra of **10**.

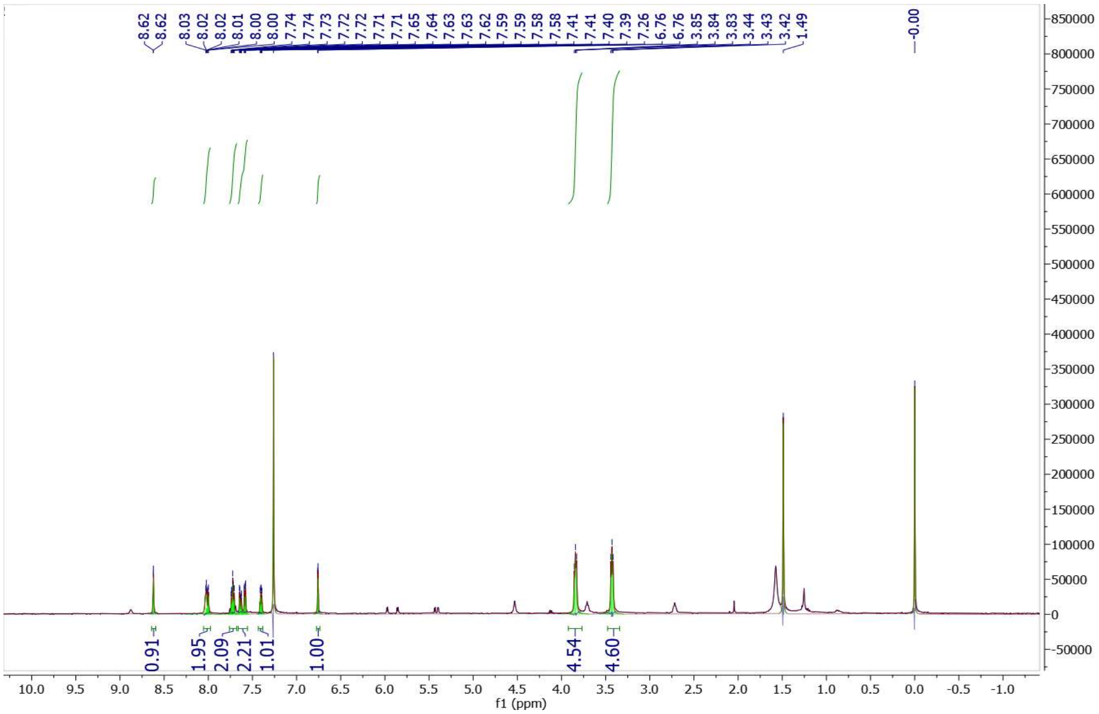
8.62
8.62
8.03
8.02
8.02
8.01
8.00
8.00
7.74
7.74
7.73
7.72
7.72
7.71
7.71
7.65
7.64
7.63
7.63
7.62
7.59
7.59
7.58
7.58
7.41
7.41
7.40
7.39
7.26
6.76
3.85
3.84
3.83
3.44
3.43
3.42
1.49
-0.00
0.91
1.95
2.09
2.21
1.01
1.00
4.54
4.60
f1 (ppm)

(a)

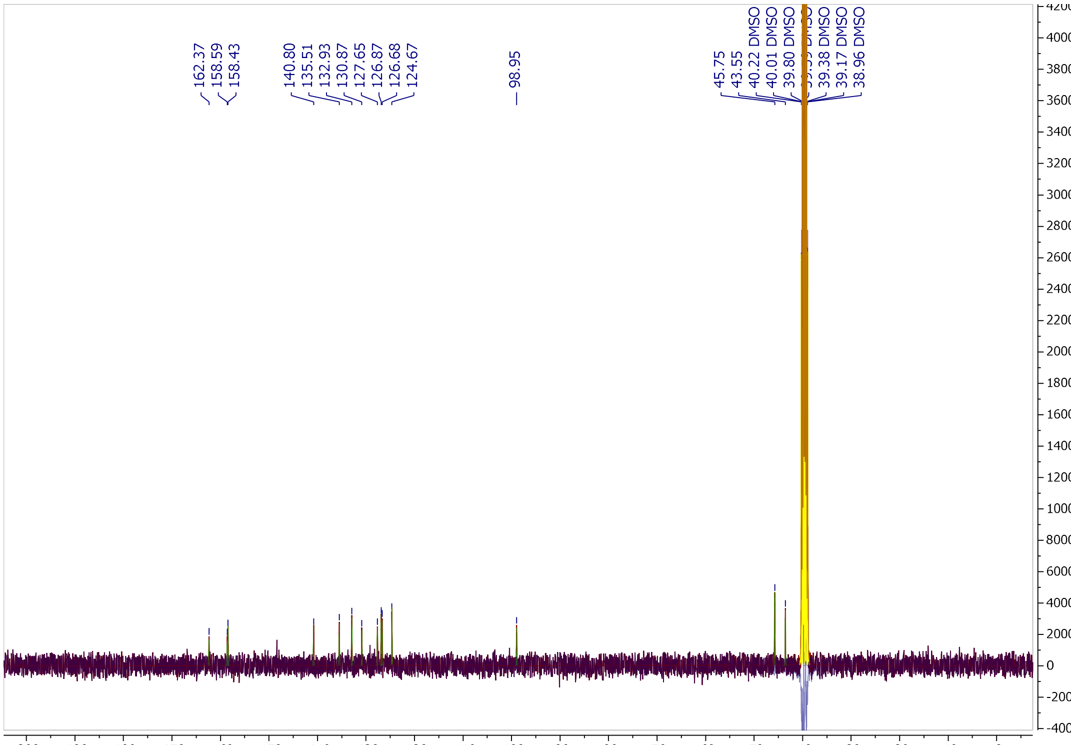
162.37
158.59
158.43
140.80
135.51
132.93
130.87
127.65
126.87
126.68
124.67
98.95
45.75
43.55
40.22 DMSO
40.01 DMSO
39.80 DMSO
39.38 DMSO
39.17 DMSO
38.96 DMSO
f1 (ppm)

(b)

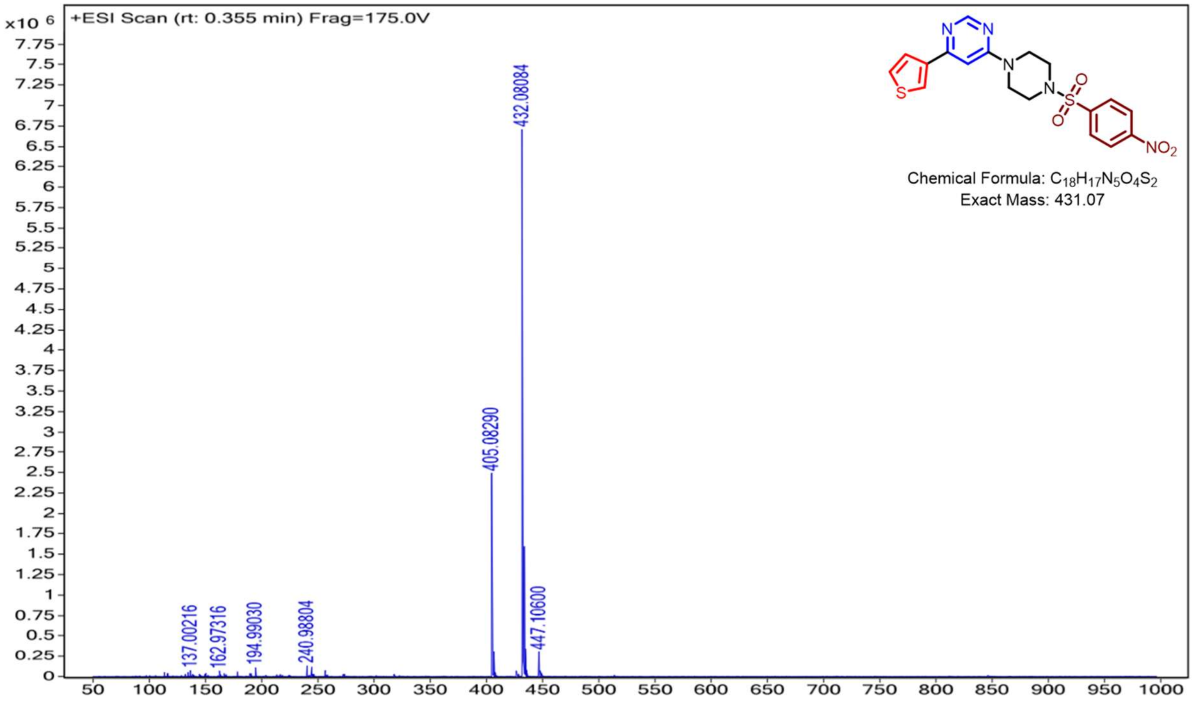

(c)

Figure SF4: (a) ^{1}H-NMR and (b) ^{13}C NMR and (c) Mass spectra of **11**.

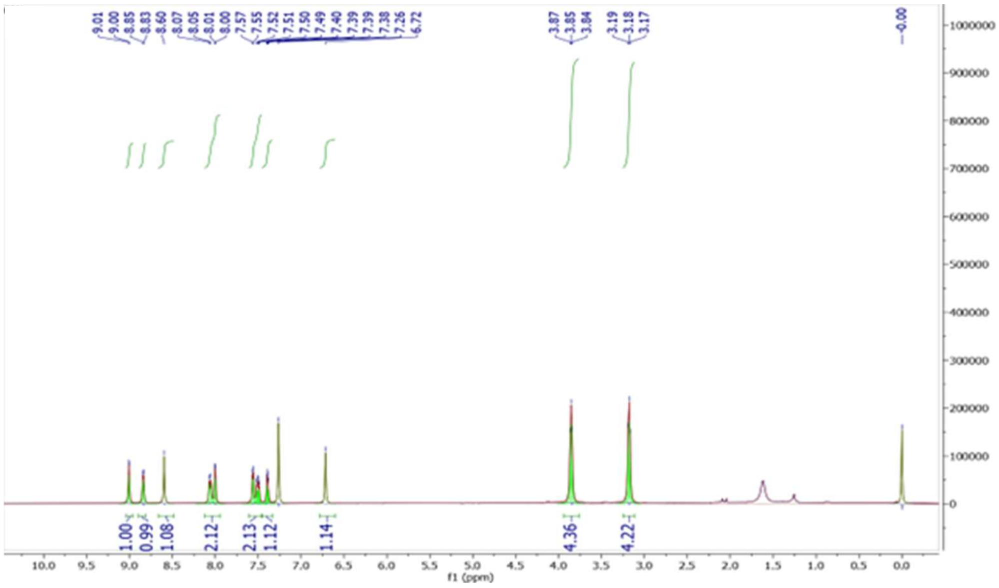
9.01
9.00
8.85
8.83
8.60
8.07
8.05
8.01
8.00
7.57
7.55
7.52
7.51
7.50
7.49
7.40
7.39
7.39
7.38
7.26
6.72
3.87
3.85
3.84
3.19
3.18
3.17
-0.00
1.00
0.99
1.08
2.12
2.13
1.12
1.14
4.36
4.22
f1 (ppm)

(a)

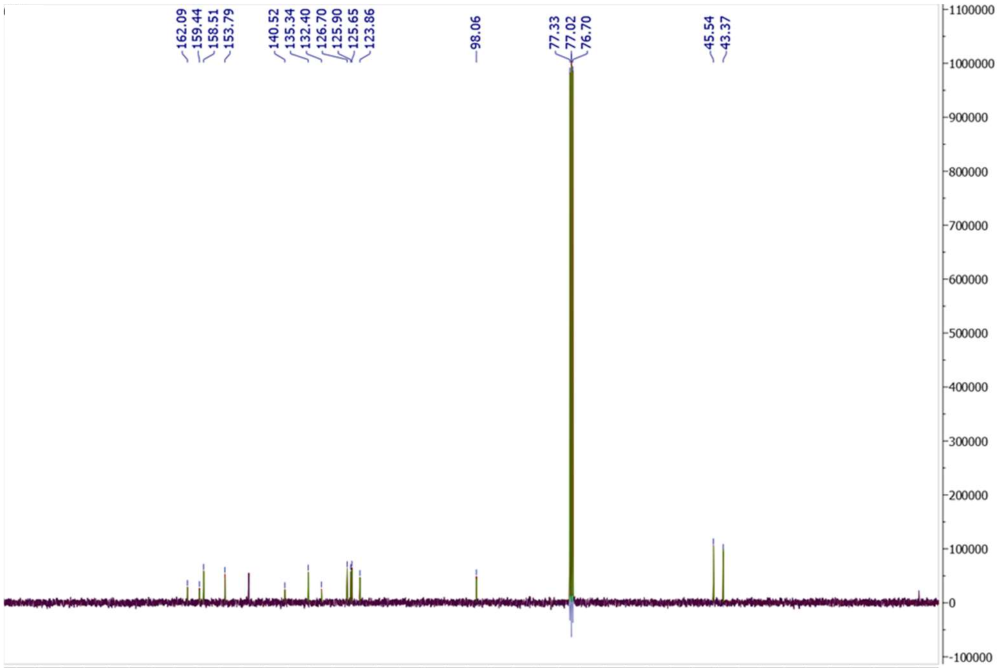
162.09
159.44
158.51
153.79
140.52
135.34
132.40
126.70
125.90
125.65
123.86
98.06
77.33
77.02
76.70
45.54
43.37
f1 (ppm)

(b)

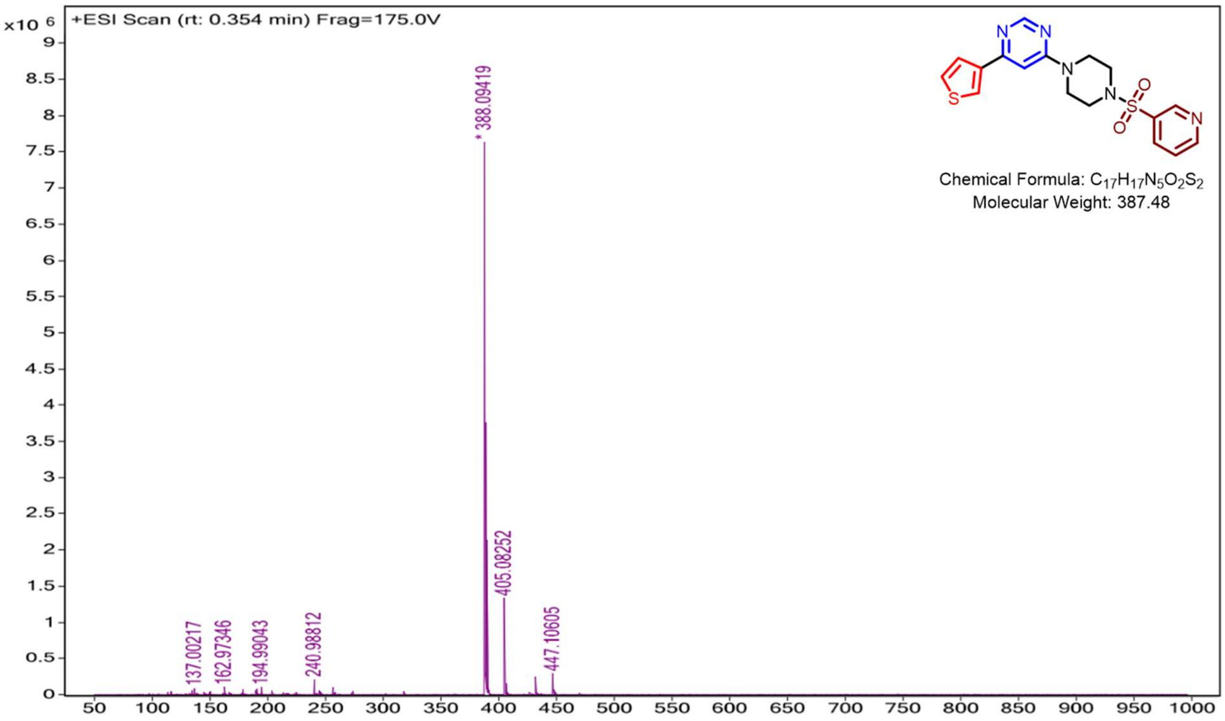

(c)

Figure SF5: (a) ^{1}H-NMR, (b) ^{13}C NMR and (c) Mass spectra of **12**.

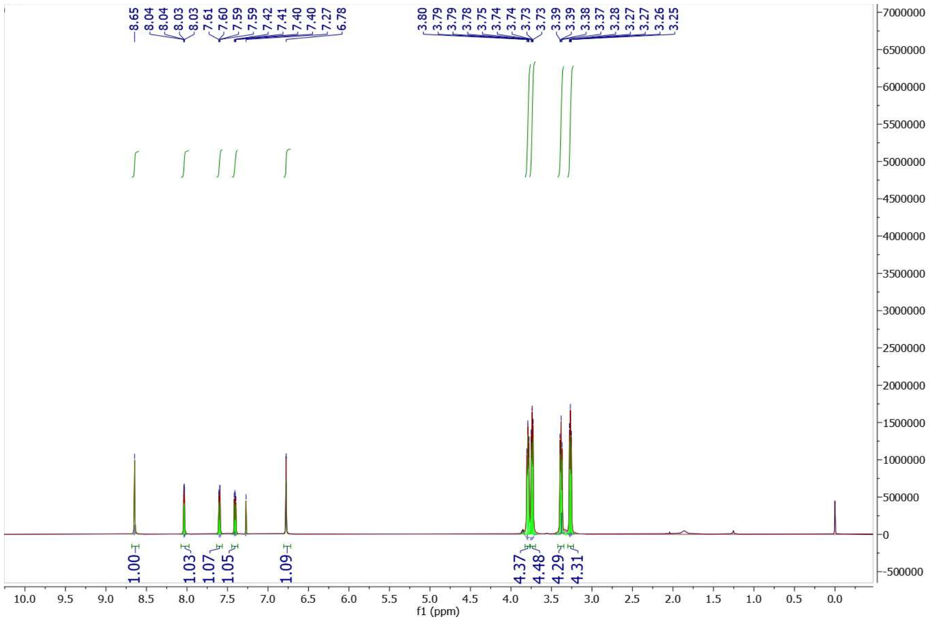

(a)

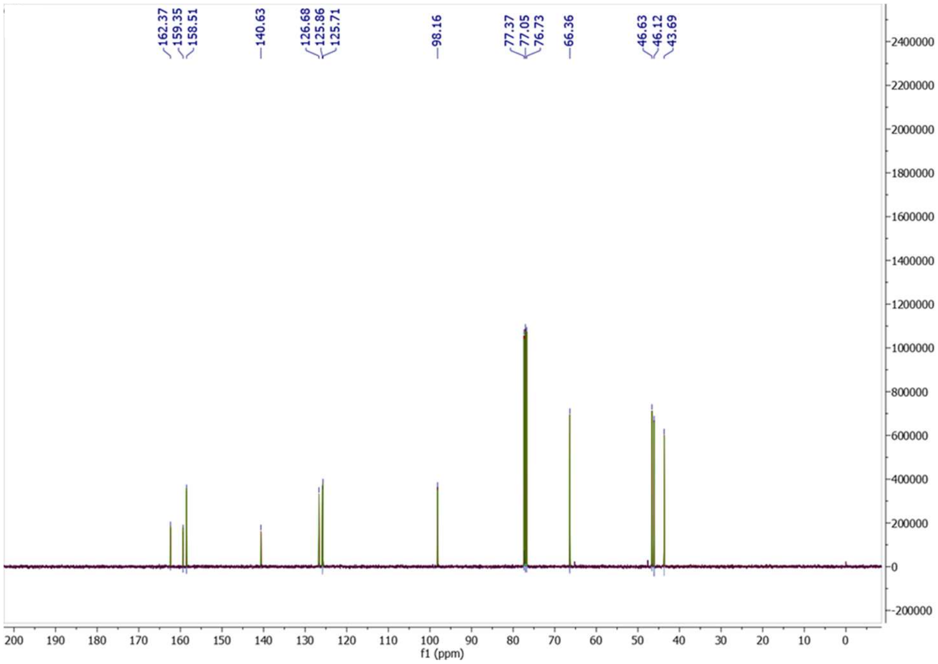

(b)

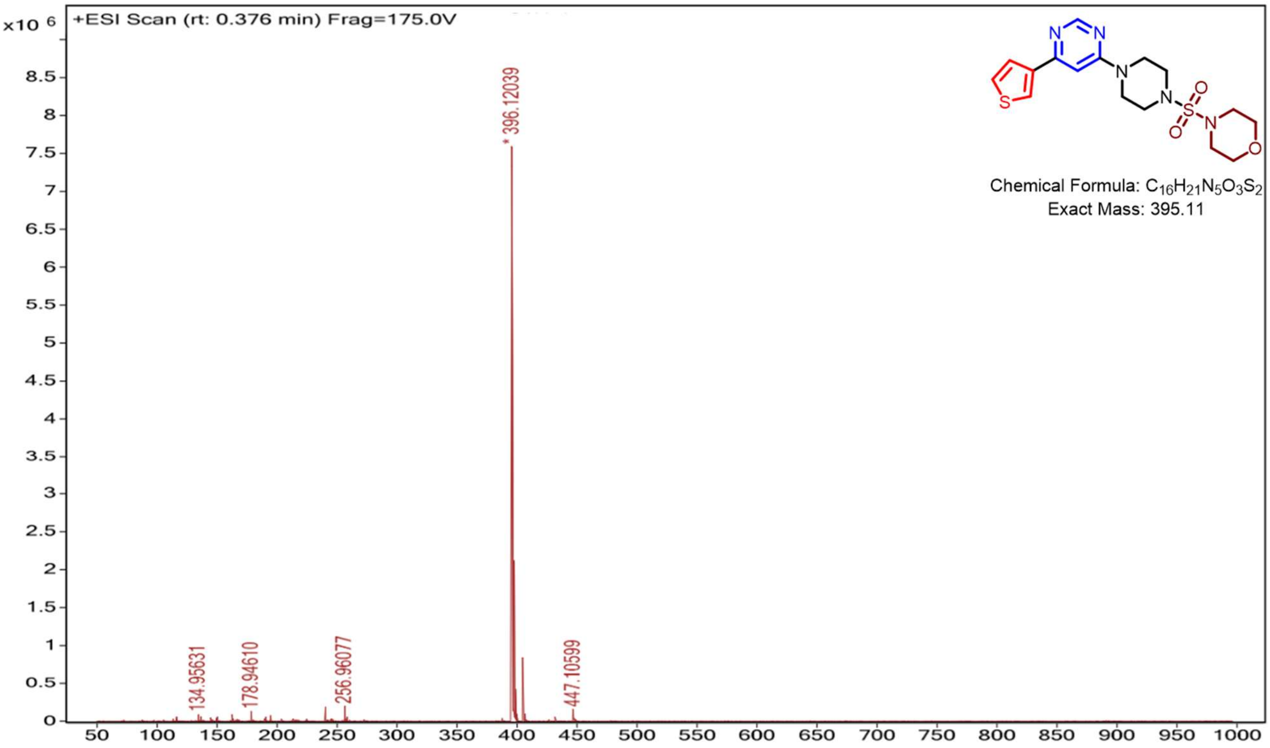

(c)

Figure SF6: (a) ^{1}H-NMR, (b) ^{13}C NMR and (c) Mass spectra of **13**.

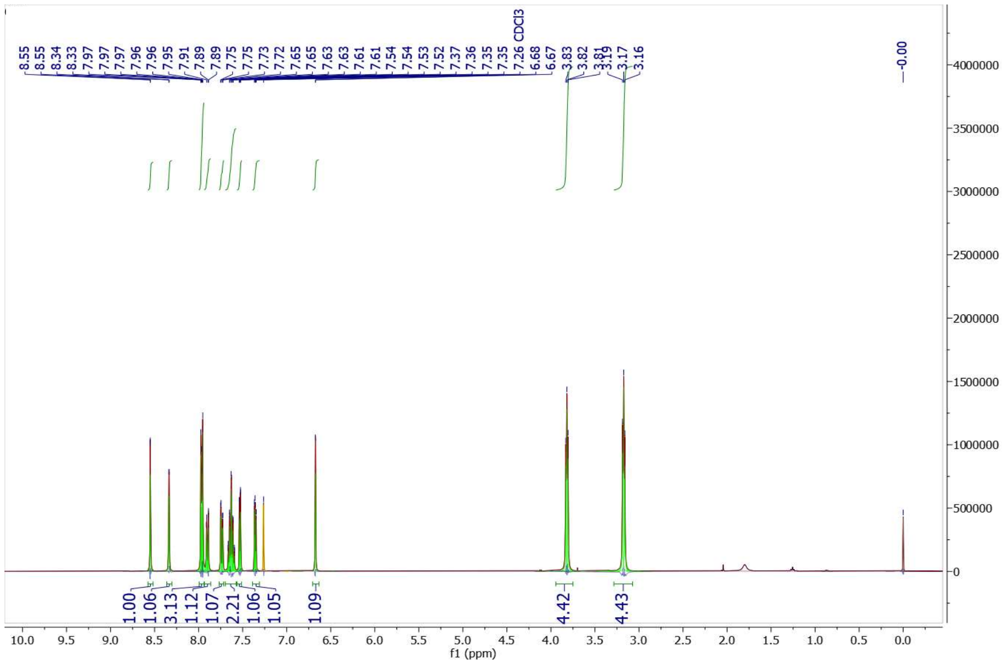

8.55 8.55 8.34 8.33 7.97 7.97 7.96 7.96 7.95 7.91 7.89 7.89 7.75 7.75 7.73 7.72 7.65 7.65 7.63 7.63 7.63 7.61 7.61 7.54 7.54 7.53 7.52 7.37 7.36 7.35 7.35 7.26 CDCl3 6.68 6.67 3.83 3.82 3.81 3.19 3.17 3.16
-0.00
1.00 1.06 3.13 1.12 1.07 2.21 1.06 1.05 1.09 4.42 4.43
f1 (ppm)

(a)

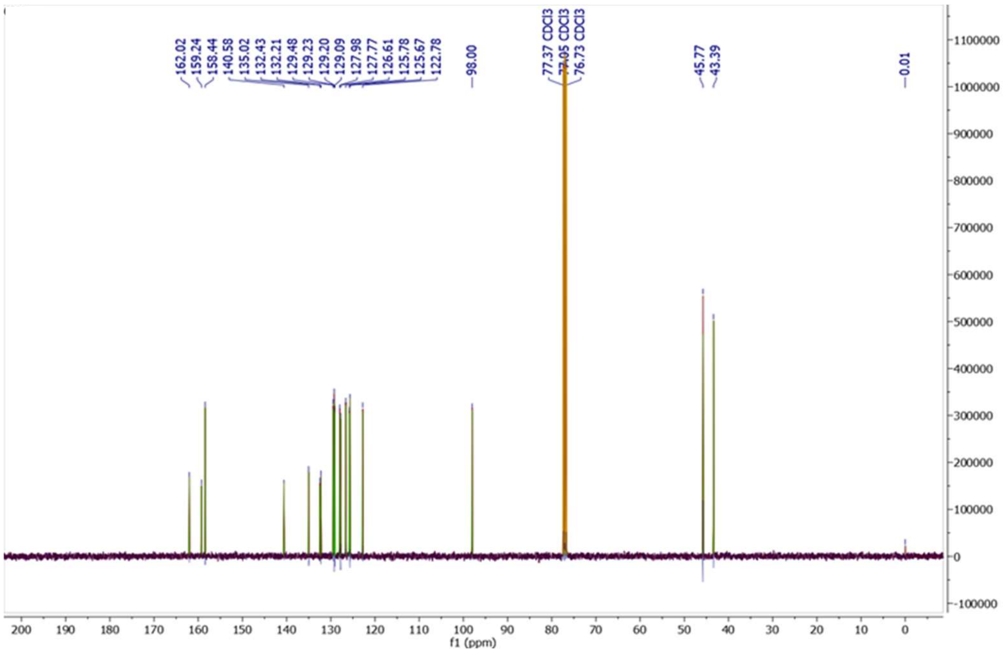

162.02 159.24 158.44 140.58 135.02 132.43 132.21 129.48 129.23 129.20 129.09 127.98 127.77 126.61 125.78 125.67 122.78
98.00
77.37 CDCl3 77.05 CDCl3 76.73 CDCl3
45.77 43.39
-0.01
f1 (ppm)

(b)

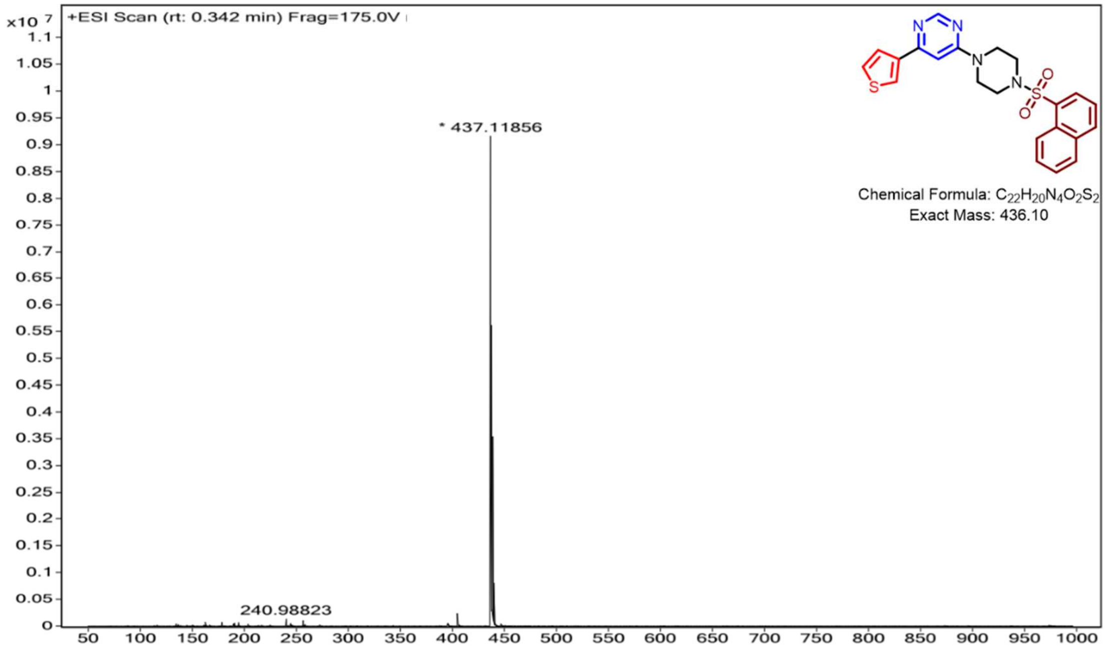

(c)

Figure SF7: (a) ^{1}H-NMR, (b) ^{13}C NMR and (c) Mass spectra of **14**.

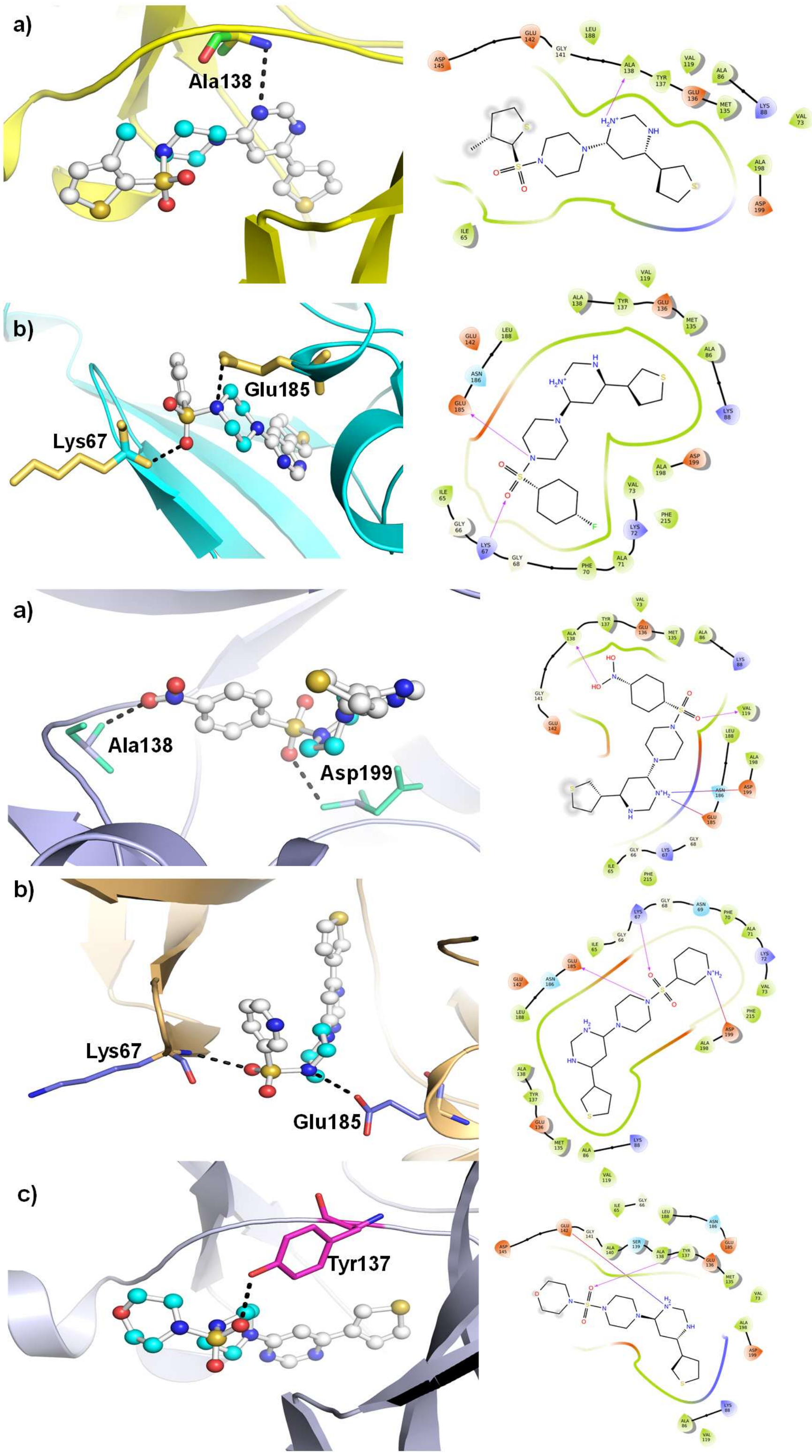

Figure SF8: The binding interactions between the target protein and the ligands were analyzed, with the protein visualized as a cartoon model, while compounds (8), (10), (11-13) are illustrated using ball-and-stick representations in panels (a), (b) and (c), respectively.

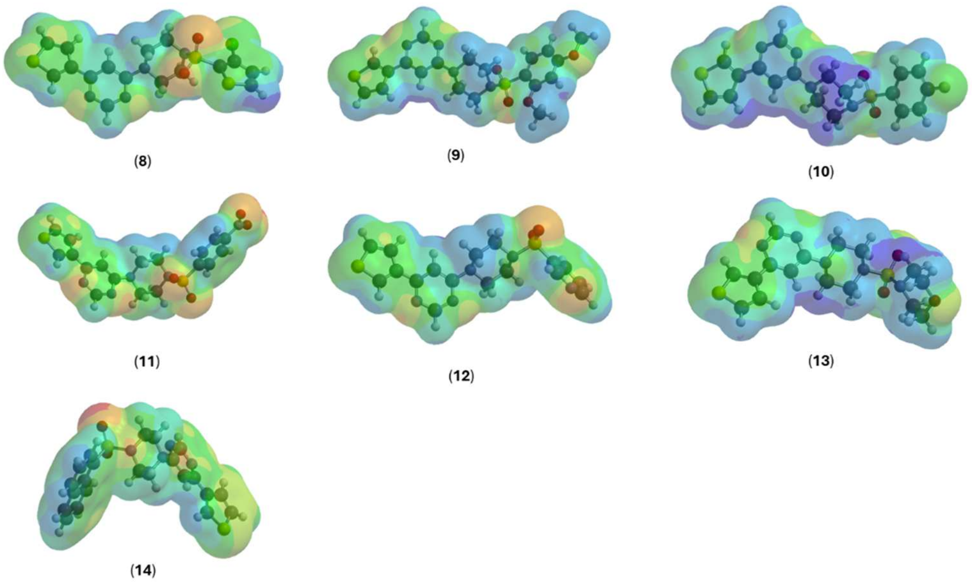

Figure SF9: DFT calculated electrostatic potential (ESP) map of compounds 8-14.

Table ST1: Toxicity prediction of the reference and screened compounds using pKCSM.[1]

Code #	Parameters									
	AT[a]	MTD[b]	hERG I[c]	hERG II[d]	ORAT[e]	CORAT[f]	Hep[g]	SS[h]	TPT[i]	MT[j]
**8**	Yes	-0.611	No	No	2.125	1.318	Yes	No	0.688	-1.25
**9**	No	-0.308	No	No	2.204	1.288	Yes	No	0.392	-1.23
**10**	No	-0.435	No	Yes	2.09	1.339	Yes	No	0.514	-1.223
**11**	Yes	-0.519	No	No	3.012	1.229	Yes	No	0.453	-1.742
**12**	Yes	-0.826	No	No	2.137	1.164	Yes	No	0.451	0.838
**13**	No	-0.875	No	No	2.279	1.146	Yes	No	0.61	1.71
**14**	No	0.308	No	No	2	1.77	Yes	No	0.341	-3.533

[a]AT = AMES Toxicity; [b]MTD = Max. Tolerated Dose (log mg/kg/day); [c]hERG I inhibitor; [d]hERG II inhibitor; [e]ORAT = Oral Rat Acute Tox (LD50, mol/kg); [f]CORAT = Chronic Oral Rat Toxicity LOAEL, log mg/kg_bw/day); [g]Hep =Hepatotoxicity; [h]SS =Skin sensitisation; [i]TPT =T. Pryiformis toxicity (log µg/L); [j]MT = Minnow toxicity (log mM)

Table ST2. Frontier molecular orbitals (FMOs) and chemical reactivity parameters of 8-14.

Code #	E_{HOMO} (eV)	E_{LUMO} (eV)	ΔE^a (eV)	I^b (eV)	A^c (eV)	χ^d (eV)	η^e (eV)	σ^f (eV^{-1})	μ^g (eV)	ω^h (eV)
**8**	–6.19	–1.47	4.72	6.19	1.47	3.83	2.36	0.211	–3.83	3.10
**9**	–5.99	–1.08	4.91	5.99	1.08	3.53	2.45	0.203	–3.53	2.53
**10**	–6.19	–1.21	4.98	6.19	1.21	3.70	2.49	0.200	–3.70	2.74
**11**	–6.43	–3.17	3.26	6.43	3.17	4.80	1.63	0.306	–4.80	7.06
**12**	–6.34	–1.57	4.77	6.34	1.57	3.95	2.38	0.209	–3.95	3.27
**13**	–6.35	–1.35	5.00	6.35	1.35	3.85	2.50	0.250	–3.85	3.10
**14**	–6.09	–1.92	4.17	6.09	1.92	4.00	2.36	0.211	–2.00	0.85

Abbreviation: Ionisation energy (I.E), electron affinity (E.A), electronegativity (χ), chemical potential (μ), chemical hardness (η), softness (σ) and electrophilicity index (ω). a = $|E_{HOMO} - E_{LUMO}|$; b = $-E_{HOMO}$; c = $-E_{LUMO}$; d = $(I + A)/2$; e = $(I - A)/2$; f = $1/2\eta$; g = $-(I + A)/2$; h = $\mu^2/2\eta$.

References

[1] D.E. Pires, T.L. Blundell, D.B. Ascher, pkCSM: predicting small-molecule pharmacokinetic and toxicity properties using graph-based signatures, J. Med. Chem. 58(9) (2015) 4066-4072.
